# Quinoline–imidazole/benzimidazole derivatives as dual-/multi-targeting hybrids inhibitors with anticancer and antimicrobial activity

**DOI:** 10.1038/s41598-022-21435-6

**Published:** 2022-10-10

**Authors:** Dumitrela Diaconu, Vasilichia Antoci, Violeta Mangalagiu, Dorina Amariucai-Mantu, Ionel I. Mangalagiu

**Affiliations:** 1 Faculty of Chemistry, Alexandru Ioan Cuza University of Iasi, 11 Carol I, 700506 Iasi, Romania; 2 Institute of Interdisciplinary Research, Department of Exact and Natural Sciences - CERNESIM Centre, Alexandru Ioan Cuza University of Iasi, 11 Carol I, 700506 Iasi, Romania

**Keywords:** Drug discovery, Chemistry

## Abstract

Two new classes of hybrid quinoline–imidazole/benzimidazole derivatives (the hybrid **QIBS** salts and **QIBC** cycloadducts) were designed and synthesized to evaluate their anticancer and antimicrobial activity. The strategy adopted for synthesis is straight and efficient, in four steps: *N*-acylation, *N*-alkylation, quaternization and a Huisgen 3 + 2 cycloaddition. The in vitro single-dose anticancer assay of forty six hybrid quinoline-benzimidazole compounds reveal that one **QIBS** salt (**11h**), has an excellent quasi nonselective activity against all type of cancer cell with an excellent PGI in the area of 90–100% and very good lethality. Three others quinoline–imidazole/benzimidazole hybrids (**8h**, **12h**, **12f**) has an excellent selective activity against some cancer cell lines: breast cancer MDA-MB-468 and Leukemia HL-60 TB). The five-dose assay screening confirms that compound **11h** possesses excellent anti-proliferative activity, with GI_50_ in the range of *nano*-molar, against some cancer cell lines: Leukemia HL-60 TB, Leukemia K-526, Leukemia RPMI-8226, Breast cancer MDA-MB-468, Lung cancer HOP-92 and Ovarian cancer IGROV1. The antibacterial assay indicates that three hybrid **QIBS** salts (**12f**, **12c**, **12d**) have an excellent activity against Gram-negative bacteria *E. coli* (superior to control Gentamicin) while against Gram-positive bacteria *S. aureus* only one compound **8i** (R_2_ = -CF3) exhibits a significant activity (superior to control Gentamicin). The MIC assay indicates that two other compounds (**11h**, **12h**) are biologically active to a very low concentration, in the range of *nano*-molar. We believe that all these excellent assets related to anticancer and antibacterial activities, make from our hybrid quinoline–imidazole/benzimidazole compounds bearing a phenyl group (R_2_ = –C_6_H_5_) in the *para* (4)-position of the benzoyl moiety a good candidate for future drug developing.

## Introduction

Cancer is one of the most merciless, serious and life-threatening disease worldwide, being characterized by the uncontrolled and rapid pathological growth of abnormal cells in the body and spread in different organs (metastasis)^1^. Based on WHO data, cancer continues to grow all around the world becoming the second leading cause of death worldwide (with over 10 million deaths in 2020) and exerting a great pressure on health systems, individuals and communities^2–5^.

Infectious diseases caused by microorganisms (especially bacteria and fungi) represent another major threat endanger human life and health^6^. In particular, overconsumption, widespread use and misuse of antimicrobials^2,6^ have caused seriously problems in the treatment of many microbial illnesses^7,8^.

In spite of the greatest advances achieved in both cancer and microbial therapy, the existing treatment suffers from some major limitations, these including drug resistance, multi-drug resistance, extensively-drug-resistance, often high toxicity levels and non specificity of drugs, high prices, etc.^7–12^. Thus, continued searching for newer and better anticancer and antimicrobial drugs remains a very important clue in medicinal chemistry.

A literature survey revealed that among heterocycles, quinoline and imidazole/benzimidazole derivatives are privileged scaffolds for the development of new drug entities. In this respect they possess a wide range of biological activities such as anticancer, antiplasmodial and antimalarial, antitubercular, antibacterial, antifungal, antiviral, anthelmintic, anti-HIV, analgesic, anticonvulsant, anti-inflammatory, antihistaminic, antipsychotic, anti-Alzheimer’s, antihypertensive, etc.^9–26^.

In continuation of our seeking for new entities with anticancer and antimicrobial activity with quinoline and imidazole/benzimidazole skeleton^21–26^, we report herein the design, synthesis and biological activity of two new class of hybrid molecules with quinoline–imidazole/benzimidazole skeleton.

## Results and discussion

### Design and synthesis

During the last few years molecular hybridization became a powerful tool in drug design and discovery offering an attractive approach to obtain better drugs for the treatment of a large variety of human diseases including cancer and microbial illness. One of the methods used for the construction of hybrid molecules combines two or more drug pharmacophores in a single multi-functional molecule using a linker chain. The main goals of this pharmacophore merging approach consist in the interaction of the resulting molecule with dual or multiple targets, amplifying the biological activity and specificity, reducing the known side effects associated with each hybrid part, reducing the drug-drug interactions^26–32^. By doing so, the hybrid drugs became more powerful and valuable than conventional classic drugs.

**Figure 1:**
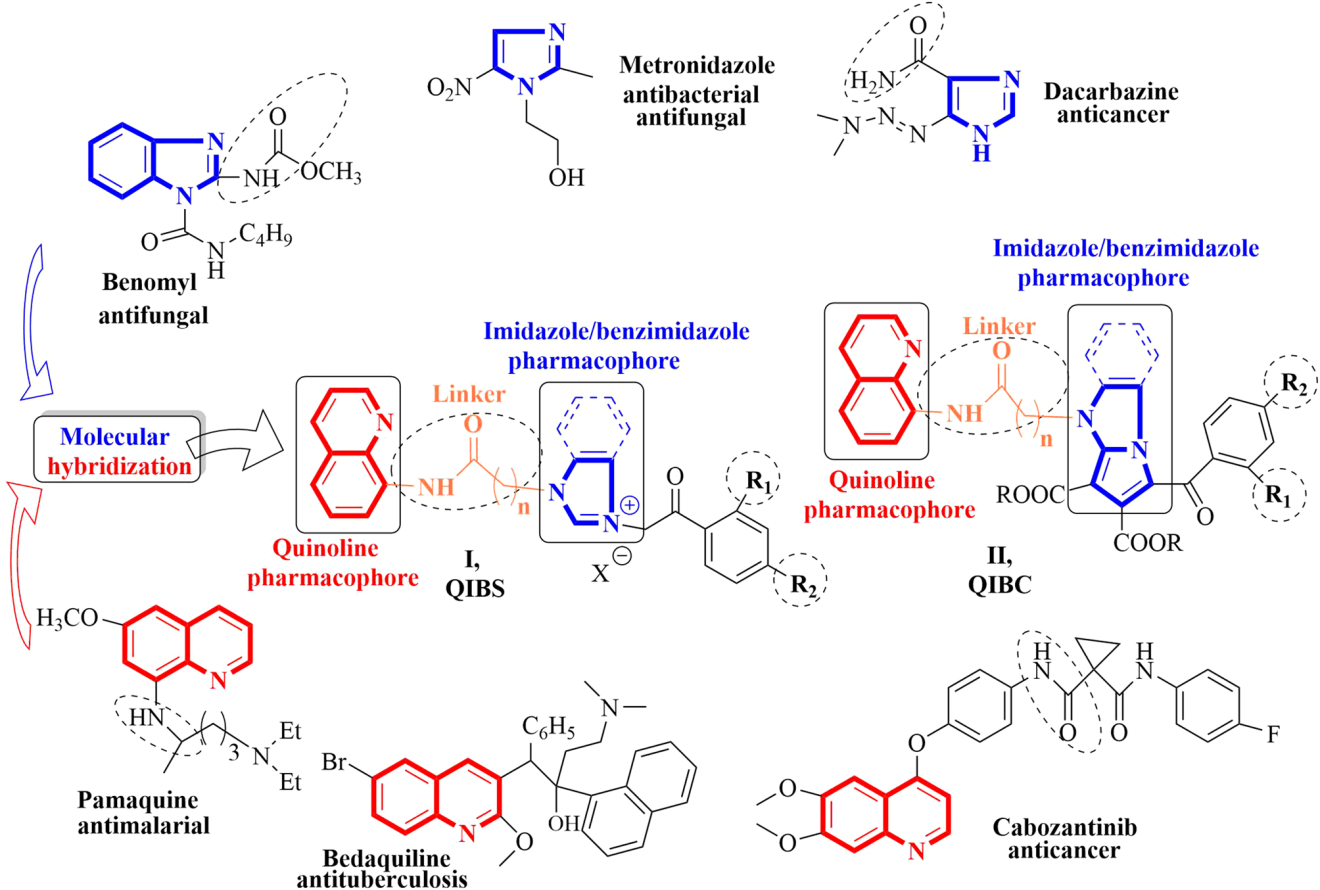
Design in the class of hybrid quinoline–imidazole/benzimidazole derivatives **QIBS** and **QIBC**.

In previous research work we successfully identify quinoline and imidazole/benzimidazole derivatives with anticancer and antimicrobial activity^22–26^. Using molecular hybridization and our previous results in the field, in the present study we aimed to obtain two new classes of hybrid molecules which combine the quinoline and imidazole/benzimidazole pharmacophores, linked by an amide-alkyl unit. A first class of merged molecule has a **q**uinoline–**i**midazole/**b**enzimidazole **s**alt structure (**I**, **QIBS**) while the second one has a **q**uinoline–**i**midazole/**b**enzimidazole **c**ycloadduct structure (**II**, **QIBC**), Fig. 1. For both classes of molecules, the two pharmacophores will assure the anticancer and antimicrobial activity while the amide-alkyl linker/spacer will realize amide bonds and a different spatial orientation (via the different length of alkyl chain) between the quinoline–imidazole/benzimidazole units. The amide-alkyl linker/spacer will be also responsible for better pharmacological properties (especially water solubility and membrane permeability) and for a more suitable interaction with the putative binding site.

In the case of **QIBS** class **I**, the introduction of a nitrogen positive charge on imidazole moiety will increase membrane permeability and water solubility, and also will lead to more potent antibacterial compounds with broadened spectrum. In equal measure we were interested to study the influence of substituents R_1_ and R_2_ from the benzoyl moieties anchored to imidazole/benzimidazole unit. The literature data reveal that a halogen atom or a phenyl ring anchored on the benzoyl moiety, especially at the *para* position of the ring, have a positive influence on both antimicrobial and anticancer activities^24,33–35^.

**Figure 2:**
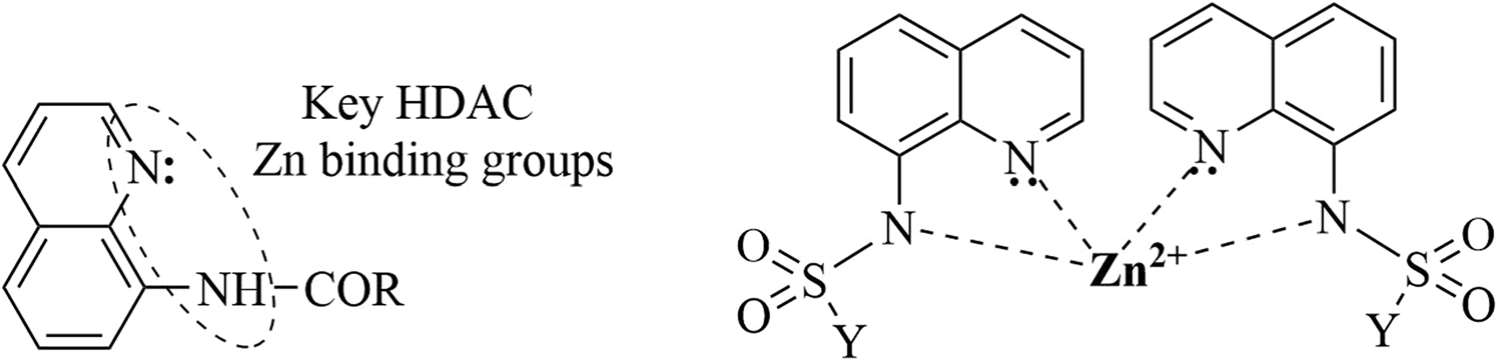
HDAC inhibitors and zinc complexes of 8-aminiquinoline derivatives.

Having in view the molecular hybridization approach used and our previously results^24,35^, we estimate as possible target (binding site) for our hybrid quinoline–imidazole/benzimidazole derivatives ATP synthase and Topoisomerase II. Also the literature data indicate zinc binding groups, amide and nitrogen from quinoline moiety, as a classic pharmacophoric model of histone deacetylases (HDAC) inhibitors^36^, Fig. 2.

As a matter of fact, the ability of amide group and nitrogen from quinoline moiety to bind zinc, is well known and experimentally proved by obtaining and fully characterized (X-ray on monocrystal including) of such of complexes, by us including^23,37^.

**Figure 3:**
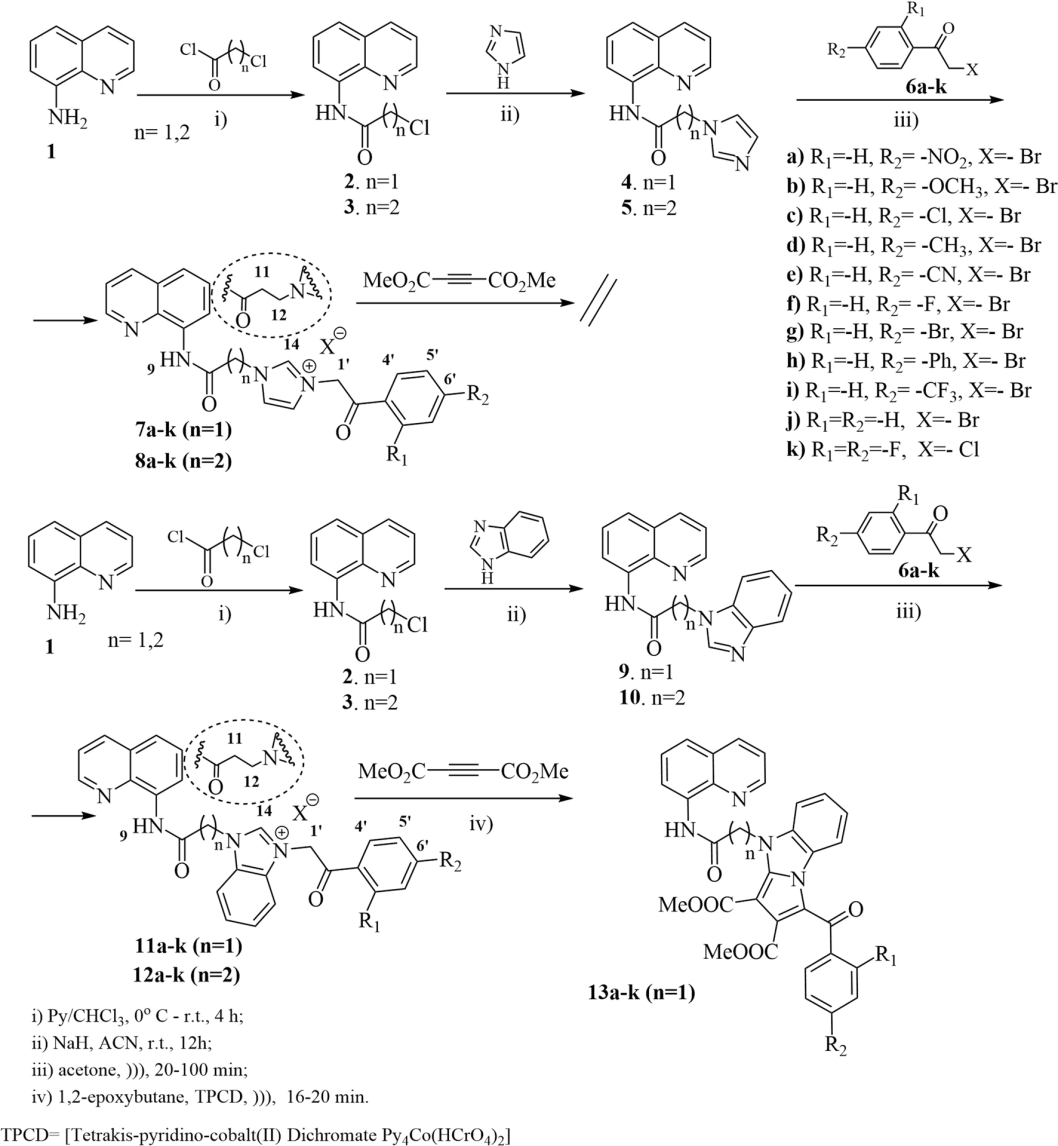
Reaction pathway to obtain **QIBS** (**7**, **8**, **11**, **12**) and **QIBC** (**13**) hybrid derivatives.

In order to obtain our **QIBS** and **QIBC** derivatives we used a similar procedure reported previously by our group^21^. The reaction pathway is straight and efficient, involving four steps indicated in Fig. 3. The initial *N*-acylation reaction of 8-aminoquinoline, is followed by an *N*-alkylation of the -NH- amino group from imidazole/ benzimidazole moiety leading to the key quinoline—imidazole/ benzimidazole intermediates, **4**, **5**, **9**, **10**. In the next step, a quaternization reaction of *N*-imidazole atom with variously activated halogenated derivatives **6a–k** (ϖ–bromo/chloro 2,4-R_1_,R_2_-substituted-acetophenones), is leading to a first class of hybrid quinoline–imidazole/benzimidazole derivatives: the salts **QIBS** derived from imidazole **7a–k** (with one methylene group as linker) and **8a–k** (with two methylene groups as linker), respectively the salts **QIBS** derived from benzimidazole **11a–k** (with one CH_2_ group) and **12a–k** (with two CH_2_ groups). In the last step imidazolium and benzimidazolium ylides, generated in situ from the corresponding **QIBS** salts **7a–k**, **8a–k** respectively **11a–k**, were treated with the activated alkyne dimethyl acetylenedicarboxylate (DMAD), when a Huisgen 3 + 2 cycloaddition take place, generating the second class of hybrid quinoline–imidazole/benzimidazole derivatives, the cycloadducts **QIBC 13a–k**, Fig. 3. It should be noted that the cycloaddition reaction with DMAD take place only in the case of benzimidazolium ylides while in the case of imidazolium ylides the reactions do not occur, leading to decomposition products.

As in related cases^23^, under conventional thermal heating (TH) the yields were low to moderate and the reactions conditions relatively harsh. As a result we perform the synthesis under ultrasounds (US) irradiation and the obtained results were much better: higher yields with about 10–20% and a significant reduction in reaction time, from hours to minutes.

The structures of the newly compounds were proven by elemental (C, H, N) and spectroscopic analysis: IR, ^1^H-NMR, ^13^C-NMR, and two-dimensional experiments 2D-COSY, 2D-HMQC, 2D-HMBC.

If we consider the most active and promising anticancer and antimicrobial compounds as representative for their series (n = 2, namely **8a**, **8b**, **8c**, **8d**, **8h** and **12a**, **12b**, **12c**, **12d**, **12h**), the most informative signals furnished by ^1^H-NMR spectra are those one of the protons from the three aliphatic methylene groups [H11, H12 (the ethylene linker), H1′ (from CH_2_–CO)], the aromatic protons H14 (from 2 position of imidazole ring), H4′, H5′ and protons from 6′ position (from benzoyl), and proton H9 from the amide functional group, Table 1.

**Table 1:** The main ^1^H-NMR spectral data for the most biologically active hybrids quinoline–imidazole/benzimidazole compounds **8** and **12**.

Compound/proton	H11	H12	H1′	H9	H14	H4′	H5′	6′ position
**8a**, *p*-NO_2_	3.34	4.65	6.13	10.37	9.20	8.26	8.43	–
**8b**, *p*-OCH_3_	3.34	4.64	6.10	10.37	9.20	8.01	7.14	3.89
**8c**, *p*-Cl	3.35	4.65	6.09	10.36	9.22	8.05	7.71	–
**8d**, *p*-CH_3_	3.34	4.64	6.04	10.37	9.21	7.93	7.43	2.41
**8h**, *p*-C_6_H_5_	3.36	4.66	6.11	10.38	9.23	8.12	7.94	
								7.46 H10′
								7.53 H9′
								7.80 H8′
**12a**, *p*-NO_2_	3.44	4.99	6.49	10.38	9.79	8.34	8.46	–
**12b**, *p*-OCH_3_	3.44	4.97	6.36	10.38	9.79	8.07	7.17	3.89
**12c**, *p*-Cl	3.44	4.98	6.42	10.38	9.79	8.12	7.73	
**12d**, *p*-CH_3_	3.43	4.97	6.39	10.38	9.79	8.01	7.46	2.43
**12h**, *p*-C_6_H_5_	3.47	5.00	6.47	10.40	9.82	8.19	7.98	
								7.47 H10′
								7.56 H9′
								7.82 H8′

The most deshielded protons are H9 (10.40–10.36 ppm, singlet) from amide functional group, which is characteristic for this functionality. The next deshielded protons are H14 (9.82–9.20 ppm, singlet) from the two positions of imidazole/benzimidazole moiety, due to the powerful deshielding effect of the positive nitrogen atom N15 and to N13 nitrogen. The protons from benzoyl moiety (H4′, H5′) appear at chemical shifts in accordance with the electronic effects exerted by the substituent from the *para* position of the benzoyl ring (8.46–7.14 ppm, doublet, *J* = 8.0 Hz). The signals of the H1′ protons from methylene group appear as a singlet, at a very low field, unusual for this type of protons, around 6.47–6.04 ppm. This is due to the powerful electron withdrawing effect of the adjacent carbonyl group and positive nitrogen atom N15 from imidazole/benzimidazole moiety. The signals of the protons from ethylene linker appear at 5.00–4.64 ppm for H12 (doublet, *J* = 6.5 Hz) respectively 3.47–3.34 ppm for H11 (triplet, *J* = 6.5 Hz), in accordance with the influence of their substituents: α-nitrogen atom N13, β-carbonyl group for H12, respective β-nitrogen atom N13, α-carbonyl group for H11. If we compare the spectra of compounds **12** (containing a benzimidazole moiety) with spectra of compounds **8** (containing a imidazole moiety), we may notice a clear influence of benzimidazole moiety related to the protons from ethylene linker (H11, H12) which consist in a deshielding process with about 0.35 ppm on H12 protons and 0.1 ppm on H11 protons, in accordance with the withdrawing effect of the heterocyclic ring.

In the ^13^C-NMR spectra of compounds **8** and **12**, the most important data are furnished by the signals corresponding to the carbonyl groups (C10 and C2′), the three carbons from the methylene groups (C11, C12, C1′), the aromatic carbons C14 (from imidazole ring) and C3′, C4′, C5′, C6′ (from benzoyl moiety), Table 2.

**Table 2:** The main ^13^C-NMR spectral data for the most biologically active hybrids quinoline–imidazole/benzimidazole compounds **8** and **12**.

Compound/carbon	C10	C2’	C11	C12	C1′	C14	C3′	C4′	C5′	C6′
**8a**, *p*-NO_2_	168.6	190.7	36.2	45.3	55.8	137.7	138.2	129.6	124.1	150.5
**8b**, *p*-OCH_3_	168.6	189.5	36.2	45.2	55.0	137.7	126.5	130.5	114.3	164.0
**8c**, *p*-Cl	168.6	190.5	36.2	45.2	55.4	137.7	132.4	130.0	129.2	139.3
**8d**, *p*-CH_3_	168.6	190.7	36.2	45.2	55.2	137.7	131.2	128.2	129.6	145.1
**8h**, *p*-C_6_H_5_	168.6	190.8	36.2	45.2	55.4	137.8	132.5	128.9	127.1	145.7
**12a**, *p*-NO_2_	168.8	190.6	35.2	43.2	53.7	143.9	138.4	129.9	124.1	150.6
**12b**, *p*-OCH_3_	168.8	189.4	35.2	43.2	52.8	144.0	126.6	130.9	114.3	164.2
**12c**, *p*-Cl	168.8	190.4	35.2	43.2	53.3	143.9	132.5	130.3	129.2	139.4
**12d**, *p*-CH_3_	168.8	190.7	35.2	43.2	53.3	144.0	130.7	129.6	128.5	145.3
**12h**, *p*-C_6_H_5_	168.8	190.7	35.2	43.2	53.2	143.9	132.5	129.2	127.1	145.7

The most deshielded signals are those one of the carbon from carbonyl ketone group (C2′) which appear around 190.7–189.4 ppm, typical for an alkyl-aryl C=O carbonyl ketone group. They are followed by the signals of the carbon from carbonyl amide group (C10, appear around 168 ppm), which is also typical for C=O carbonyl amide group. The heteroaromatic carbons C14 from imidazole ring appear around 144 ppm in compounds **12** (containing a benzimidazole moiety) respectively 137 ppm in compounds **8** (containing a imidazole moiety), in accordance with the powerful deshielding effect of the two adjacent nitrogen atoms from imidazole ring. The three carbons from the methylene groups (C11, C12, C1′) appear around 55–53 ppm (C1′: α-ketone, α-positive nitrogen), 45–43 ppm (C12: α-nitrogen atom N13, β-carbonyl group), 36–35 ppm (C11: β-nitrogen atom N13, α-carbonyl group).

The aromatic carbons from benzoyl moiety (C3′, C4′, C5′, C6′) appear at chemical shifts in accordance with the electronic effects exerted by the substituent from the *para* position of the benzoyl ring.

All the remaining signals from NMR spectra are in accordance with the proposed structures. See also Experimental part for the ^1^H- and ^13^C-NMR spectra for compounds **8a**, **8b**, **8c**, **8d**, **8h**, **12a**, **12b**, **12c**, **12d**, **12h**.

### Anticancer activity

The in vitro anticancer assay was performed at the National Cancer Institute (NCI, USA), under the Developmental Therapeutics Program (DTP). The 60 created cell lines represent nine human cancers: breast, central nervous system, colon, kidney, leukemia, lung, melanoma, ovary, and prostate. The cancer cell lines grewup according to the NCI standard protocols. The DTP screens include the NCI 60 cell line screen, utilizing 60 different human tumor cell lines in accordance with the protocol of the NCI^38–40^. The screening is beginning with the evaluation of all compounds against the 60 cell lines at a single-dose of 10 μM^38^. The output from the single dose screen is reported as a mean graph and is available for analysis by the COMPARE program (See: http://dtp.nci.nih.gov/docs/compare/compare.html). The results are expressed as "Percentage Growth Inhibition" (PGI) term, and represent growth relative to the no-drug control, and relative to the time zero number of cells. This allows detection of both growth inhibition (values between 0 and 100) and lethality (values less than 0). For example, a value of 40 would mean 60% growth inhibition while a value of -40 would mean 40% lethality (more information could be found to Supporting Information data of this article).

Forty six of the synthesized compounds were selected and tested by the NCI for the primary single dose assay (10^–5^ M), the obtained results being presented at Supporting Information data (in Supplementary Table 1 (**QIBS** salts, **7a, b, c, d, e, f, h, k** and **11a, b, c, d, e, f, g, h, k**), Supplementary Table 2 (**QIBS** salts, **8a, b, c, d, e, f, g, h, i, j, k**), Supplementary Table 3 (**QIBS** salts, **12a, b, c, d, e, f, g, h, i, j, k**), Supplementary Table 4 (**QIBC** cycloadducts **13a, c, d, e, f, g, h**). In Table 3 are summarized the results of the NCI 60 anticancer primary single-dose assay for the most active compounds.

**Table 3:** Results of the in vitro NCI 60 human cancer cell lines single-dose assay for the most active hybrid quinoline–imidazole/benzimidazole compounds (**11h**, **8h**, **12h**, **12f**, **7h**, **11a** and **11b**), expressed as percentage growth inhibition (PGI%, μM).

Cell type	Compound/growth inhibition percent (PGI%)						
	**11h**	**8h**	**12h**	**12f**	**7h**	**11a**	**11b**
**Leukemia**							
CCRF-CEM	**90**	22	64	32	36	11	16
HL-60 (TB)	***100**** (37)*^*b*^	**87**	***100****(42)*^*b*^	64	**86**	39	73
K-562	**88**	55	78	42	50	62	49
MOLT-4	**89**	16	53	35	27	8	10
RPMI-8226	***100**** (30)*^*b*^	66	**97**	75	67	**90**	**81**
SR	***100**** (17)*^*b*^	21	**91**	21	47	39	23
**Non-small cell lung cancer**							
A549/ATCC	41	6	–	–	28	**86**	0
EKVX	53	19	39	11	24	27	6
HOP-62	57	0	6	15	22	5	10
HOP-92	***100**** (4)*^*b*^	–	–	–	27	73	61
NCI-H226	50	17	43	22	34	20	8
NCI-H23	77	19	40	17	5	50	17
NCI-H322M	23	8	18	7	13	5	3
NCI-460	78	9	45	12	58	23	3
NCI-H522	**98**	53	53	33	26	53	56
**Colon cancer**							
COLO 205	***100**** (61)*^*b*^	28	56	45	36	75	40
HCC-2998	**94**	32	62	14	49	50	30
HCT-116	**89**	16	44	47	38	63	17
HT29	**93**	30	49	42	41	60	37
KM12	***100**** (31)*^*b*^	64	**86**	34	69	22	13
SW-620	64	15	35	8	16	46	7
**CNS cancer**							
SF-268	**80**	18	45	32	35	35	31
SF-295	22	0	–	–	0	0	1
SF-539	77	20	25	14	21	13	19
SNB-19	**93**	40	59	39	43	63	32
SNB-75	***100**** (6)*^*b*^	55	59	35	37	71	65
U251	**80**	40	58	31	42	37	14
**Melanoma**							
LOX IMVI	***100**** (45)*^*b*^	18	43	16	31	14	10
MALME-3M	**92**	–	–	–	16	19	14
M14	72	18	51	21	21	22	16
MDA-MB-435	**93**	13	68	22	18	40	18
SK-MEL-2	**81**	10	35	19	26	60	26
SK-MEL-28	79	33	62	40	31	22	31
SK-MEL-5	***100**** (35)*^*b*^	19	**80**	67	66	5	3
UACC-257	**90**	60	77	53	41	52	63
UACC-62	***100**** (32)*^*b*^	42	**85**	45	32	31	43
**Ovarian cancer**							
IGROV1	***100**** (38)*^*b*^	64	**90**	60	**73**	29	42
OVCAR-3	***100**** (1)*^*b*^	49	72	45	50	62	58
OVCAR-4	**90**	**86**	76	48	68	**80**	76
OVCAR-5	38	9	19	21	10	7	10
OVCAR-8	**80**	18	48	14	24	7	3
NCI/ADR-RES	4	–	–	–	3	0	0
SK-OV-3	50	26	26	12	20	0	0
**Renal cancer**							
786–0	56	3	29	0	5	6	6
ACHN	10	2	12	0	12	7	1
CAKI-1	56	–	–	–	0	16	4
RXF 393	17	–	23	1	0	3	6
SN12C	**86**	10	31	8	15	71	17
TK-10	12	13	47	7	0	0	4
UO-31	33	2	18	12	6	35	21
**Prostate cancer**							
PC-3	**88**	45	70	37	46	62	33
DU-145	64	10	22	4	24	15	2
**Breast cancer**							
MCF7	**88**	42	**81**	32	50	70	25
MDA-MB-231/ATCC	***100**** (7)*^*b*^	35	51	43	27	50	11
HS 578T	67	29	48	26	25	26	12
BT-549	***100**** (9)*^*b*^	43	47	31	43	65	56
T-47D	**90**	58	**80**	47	60	67	66
MDA-MB-468	***100**** (6)*^*b*^	***100****(26)*^*b*^	***100**** (32)*^*b*^	***100**** (17)*^*b*^	**92**	**91**	**86**

The number reported for the one-dose assay, percentage growth inhibition (PGI), is growth relative to the no-drug control, and relative to the time zero number of cells; ^b^ Cytotoxic effect; lethality (L) percent is represented in brackets; the most active compounds are highlighted in bold and italics.

From Table 3 we may notice the most active compound is **QIBS** salt, **11h** (hybrid quinoline-benzimidazole salt, n = 1), with a quasi nonselective activity against all type of cancer cell (except renal and prostate) with an excellent PGI in the area of 90–100% and very good lethality. Another excellent result is the selectivity against some cancer cell lines exerted by some hybrid quinoline-imidazole/benzimidazole salts with ethylene linker (n = 2): **12h** on two cancer lines [Breast cancer MDA-MB-468 (PGI = 100% and L = 32%) and Leukemia HL-60 TB (PGI = 100% and L = 42%)], respectively to one cancer line **12f** [Breast cancer MDA-MB-468 (PGI = 100% and L = 17%)] and **8h** [Breast cancer MDA-MB-468 (PGI = 100% and L = 26%)].

The results obtained for the hybrid quinoline–imidazole/benzimidazole cycloadducts (**13a, c, d, e, f, g, h**) were disappointing, with low value of PGI (see Table 4 from Supporting Information data).

Compound **11h** which had the best growth inhibition profile among the tested compounds, was selected by NCI for detailed studies at five different concentrations (0.01, 0.1, 1, 10 and 100 μM) in the five-dose assay studies^38–40^, selected results being presented in Table 4.

**Table 4:** Results of the 5-dose in vitro human cancer cell growth inhibition (data obtained from NCI’s in vitro 60 cell 5-dose screening) assay for compound **11h**.

Cell type	Compound/GI_50_ (μM)^a^	Cell type	Compound/GI_50_ (μM)^a^	Cell type	Compound/GI_50_ (μM)^a^
	**11h**		**11h**		**11h**
*Leukemia*		*Non-small cell lung cancer*		*Colon cancer*	
CCRF-CEM	1.57	549/ATCC	3.02	COLO 205	1.59
HL-60 (TB)	**0.428**	EKVX	1.63	HCC-2998	1.41
K-562	**0.639**	HOP-62	1.78	HCT-116	1.69
MOLT-4	1.75	HOP-92	**0.677**	HT29	1.28
RPMI-8226	**0.911**	NCI-H226	2.02	KM12	1.23
SR	1.73	NCI-H23	1.52	SW-620	1.62
		NCI-H322M	2.80		
		NCI-460	1.78		
		NCI-H522	1.53		
*CNS cancer*		*Melanoma*		*Ovarian cancer*	
SF-268	1.51	LOX IMVI	1.54	IGROV1	**0.785**
SF-295	1.73	MALME-3 M	1.53	OVCAR-3	1.09
SF-539	1.62	MDA-MB-435	1.65	OVCAR-4	1.06
SNB-19	1.46	SK-MEL-28	1.51	OVCAR-5	1.50
U251	1.43	SK-MEL-5	1.73	OVCAR-8	1.63
		UACC-257	1.28	NCI/ADR-RES	1.87
		UACC-62	1.40	SK-OV-3	1.72
*Renal cancer*		*Prostate cancer*		*Breast cancer*	
786–0	1.88	PC-3	1.03	MCF7	1.50
ACHN	3.06	DU-145	2.22	HS 578 T	1.70
CAKI-1	2.18			BT-549	1.56
RXF 393	1.11			T-47D	1.21
SN12C	1.60			MDA-MB-468	**0.590**
TK-10	2.01				

^a^GI_50_—the molar concentration of tested compound causing 50% growth inhibition of tumor cells. Determined at five concentration levels (100, 10, 1.0, 0.1 and 0.01 μM); The most active compounds are highlighted in bold.

The in vitro five-dose assay screening results revealed that compound **11h** possesses excellent anti-proliferative activity, with GI_50_ in the range of *nano*-molar, against some cancer cell lines: Leukemia HL-60 TB (428 nM), Leukemia K-526 (639 nM), Leukemia RPMI-8226 (911 nM), Breast cancer MDA-MB-468 (590 nM), Lung cancer HOP-92 (677 nM) and Ovarian cancer IGROV1 (785 nM). Compound **11h** possesses also a good to moderate activity with GI_50_ values ranging from 1 to 3 μM against the remaining cancer cells from all nine sub-panels.

From SAR point of view, the following remarks could be done for hybrid quinoline–imidazole/benzimidazole compounds with anticancer activity:

- the most important factor that influence the biological activity is the anchored substituent from the *para (4)-*position of the benzoyl moiety, compounds with a phenyl (–C_6_H_5_) group having the highest and the most interesting activity. The presence of a fluoro (–F), nitro (–NO_2_), and methoxy (–OCH_3_) moiety are also useful for activity;
- the hybrid compounds with benzimidazole moiety (**11** and **12**) are more active than those one with a imidazole structure (**7** and **8**);
- the length of alkyl chain linker betwen quinoline and imidazole/benzimidazole moieties, influence selectivity of compounds: increasing size of alkyl length (from one methylene to two methylene groups) increase substantially the selectivity. Thus, while the benzimidazole compound **11h** (with one methylene, n = 1) have a high quasi nonselective anticancer activity, the benzimidazole compound **12h** (with two methylene, n = 2) have a high selective anticancer activity. We presume that this behaviour is related to the interaction with the receptor situs;
- the **QIBS** salts compounds are more active than **QIBC** cycloadducts, which confirms our initial hypothesis that the introducing of a nitrogen positive charge on imidazole moiety will increase membrane permeability and water solubility, and also will lead to more potent biologically active compounds.

### Antimicrobial activity

The in vitro antibacterial and antifungal activities of the hybrid quinoline–imidazole/benzimidazole compounds was determined by the Kirby-Bauer disk diffusion method^41^. The method is using μler Hinton nutrient agar medium for antibacterial assay and Sabouraud nutrient agar medium for antifungal assay. The in vitro antibacterial activity was evaluated against Gram-positive bacteria *Staphylococcus aureus ATCC 25923* and Gram-negative *Escherichia coli ATCC 25922* and the antifungal activity against fungus *Candida albicans* ATCC 10231. Gentamicin was used as positive control (C+) for *S. aureus* and *E. coli* and Nystatin for *C. albicans*. The negative control (C−) consists of sterile filter paper disks (with no antimicrobial compounds) inoculated with DMSO 3%. The obtained results are expressed as diameters of inhibition zones (mm), the larger diameter is, the most active compounds are. The obtained results are presented at Supporting Information data (in Supplementary Tables 5–8). In Table 5 below are summarized the results of the antibacterial and antifungal assay for the most active compounds.

**Table 5:** The antibacterial activity for assay for the most active hybrid quinoline–imidazole/benzimidazole compounds (**12f**, **12c**, **12d**, **11f**, **11d**, **8h**, **8d, 7d**, **7j**, **7g**, **7i** and **8i**), determined by disk diffusion assay.

Strain	Compound											
	Diameter of inhibition zone (mm)											
*E. coli*	C+	**12f**	**12c**	**12d**	**11f**	**11d**	**8h**	**8d**	**7d**	**7j**	**7g**	**7i**
	12 ± 1.1	24 ± 1.7	20 ± 2	20 ± 1.8	18 ± 1	17 ± 1.4	18 ± 2	17 ± 0.7	18.5 ± 1	18.5 ± 1	16 ± 2.3	17 ± 1
*S.aureus*	C +	**8i**										
	14 ± 1.4	20 ± 1.3										

*E. coli Escherichia coli ATCC 25922, S. aureus Staphylococcus. aureus ATCC 25923, X* ± *SD mean of three mesearurements* ± *standard deviation.*

*C+* Gentamicin for *S. aureus* and *E. coli.*

The data from Table 5 reveals that some of our hybrid quinoline–imidazole/benzimidazole compounds proved to be effective against the tested bacterial strains. Our hybrid compounds manifest a much pronounced antibacterial activity against gram-negative bacteria *E. coli* comparative with gram-positive bacteria *S. aureus*, three compounds (**12f**, **12c**, **12d**) being extremely active (having a diameter of zone inhibition up to 20 mm, superior to control Gentamicin with about 8–12 mm), while seven others (**11f**, **11d**, **8h**, **8d**, **7j**, **7i**, **7d**) are very active (having a diameter of zone inhibition superior to control Gentamicin with more than 5 mm). Against gram-positive bacteria *S. aureus* only one compound **8i** (R_2_ = − CF_3_) manifest a significant activity, superior to control Gentamicin with 6 mm.

Against fungus *C. albicans* the tested hybrid quinoline–imidazole/benzimidazole derivatives proved to be inactive.

From SAR point of view, the following remarks could be done for hybrid quinoline–imidazole/benzimidazole compounds with antibacterial activity:

- presence of a hybrid quinoline–imidazole/benzimidazole moiety have a beneficial influence for antibacterial activity against gram-negative bacteria *E. coli*, lesser influence against gram-positive bacteria *S. aureus* and no influence against fungus;
- the most important factor that influence the biological activity within the hybrid quinoline–imidazole/benzimidazole derivatives, is the presence of a benzimidazole moiety into the hybrid structure;
- another important factor that influence the antibacterial activity is the anchored substituent from the *para (4)-*position of the benzoyl moiety. The presence of classical isosteres atoms/groups fluoro (–F), chloro (–Cl) and methyl (–CH_3_) or nonclassical isosteres groups trifloromethyl (–CF_3_), are leading to very active compounds [**12f** (R_2_ = –F), **12c** (R_2_ = –Cl), **12d** (R_2_ = –Me)] respectively to active compounds [**11f** (R_2_ = –F), **11d** (R_2_ = –Me), **8i** (R_2_ = –CF_3_), **8d** (R_2_ = –Me), **7i** (R_2_ = –CF_3_), **7d** (R_2_ = –Me)].
- the length of alkyl chain linker between quinoline and imidazole/benzimidazole moieties, seems to have no significant influence on activity.

In the next step of antimicrobial assay, the minimum inhibitory concentration (MIC) of the hybrid quinoline–imidazole/benzimidazole compounds were determined, using the standardized broth microdilution assay procedure^42–46^. The resulted MIC value is defined as the lowest concentration of the antimicrobial compounds under investigation, which prevents visible growth of the tested microorganism. The obtained results are presented at Supporting Information data (in Tables 9–12). In Table 6 below are summarized the results of the antibacterial and antifungal MIC assay for the most active compounds.

**Table 6:** The minimum inhibitory concentration (MIC) for the most active hybrid quinoline–imidazole/benzimidazole compounds (**11h**, **12h**, **11i**, **11g**, **11f**, **7h**).

Strain	Compound				
	MIC (μg/mL)				
*S. aureus*	C+	**11h**	**12h**	**11i**	**11g**
	0.5	0.00001	0.0003	0.009	0.009
*E. coli*	C+	**11h**	**12h**	**11f**	**7h**
	0.25	0.004	0.004	0.009	0.001

*S. aureus Staphylococcus. aureus ATCC 25923, E. coli Escherichia coli ATCC 25922*, *C+* Gentamicin for *S. aureus* and *E. coli.*

The data from Table 6, indicate that two hybrid compounds (with phenyl group (R_2_ = –C_6_H_5_) in the *para (4)-*position of the benzoyl moiety) are active to a very low concentration, in the range of *nano*-molar against *Staphylococcus aureus*, having a MIC of 0.01 ng/mL in the case of **11h**, respectively 0.3 ng/mL in the case of **12h**. These two hybrid compounds are also active to a low concentration against *Escherichia coli*, in the range of *micro*-molar, having a MIC of 0.004 µg/mL each. Significant results were obtained against *S. aureus* for compounds **11g** and **11i** (with a MIC of 0.009 µg/mL) and against *E. coli* for compounds **11f** and **7h** (with a MIC of 0.009 µg/mL respectively 0.001 µg/mL).

## Conclusions

In summary, we report herein the design, synthesis, anticancer and antimicrobial activity of two new classes of hybrid quinoline–imidazole/benzimidazole derivatives: the salts **QIBS** (derived from imidazole and benzimidazole) respectively the cycloadducts **QIBC**. The reaction pathway is straight and efficient, involving four steps: *N*-acylation, *N*-alkylation, quaternization and a Huisgen 3 + 2 cycloaddition. Forty six of the synthesized compounds were selected and tested by the NCI for the in vitro primary single dose anticancer assay and one compound for the five-dose assay. One hybrid quinoline-benzimidazole salt, **11h**, has an excellent quasi nonselective activity against all type of cancer cell (except renal and prostate) with an excellent PGI in the area of 90–100% and very good lethality. Three other quinoline–imidazole/benzimidazole hybrids has an excellent selective activity against some cancer cell lines: **12h** on two cancer lines (Breast cancer MDA-MB-468 and Leukemia HL-60TB), **12f** on Breast cancer MDA-MB-468 and **8h** also on Breast cancer MDA-MB-468. The five-dose assay screening confirm that compound **11h** possesses excellent anti-proliferative activity, with GI_50_ in the range of *nano*-molar, against some cancer cell lines: Leukemia HL-60 TB, Leukemia K-526, Leukemia RPMI-8226, Breast cancer MDA-MB-468, Lung cancer HOP-92 and Ovarian cancer IGROV1. The SAR correlation related to anticancer activity reveals interesting data related to the influence of heterocycle (a benzimidazole moiety is favourable), length of alkyl chain linker (increasing the size of alkyl length affect substantially the selectivity) and substituent from the *para (4)-*position of the benzoyl moiety, compounds with a phenyl (–C_6_H_5_) group having the highest and the most interesting activity. Overall, the hybrid **QIBS** salts are more active than **QIBC** cycloadducts. Three hybrid **QIBS** salts (**12f**, **12c**, **12d**) have an excellent antibacterial activity against gram-negative bacteria *E. coli* (superior to control Gentamicin). Against gram-positive bacteria *S. aureus* one compound **8i** (R_2_ = –CF_3_) manifest a significant activity (superior to control Gentamicin) and the MIC assay indicate that two other compounds (**11h**, **12h**) are active to a very low concentration, in the range of *nano*-molar. Overall, the hybrid **QIBS** salts manifest a much pronounced antibacterial activity against gram-negative bacteria *E. coli* comparative with gram-positive bacteria *S. aureus*. Against fungus *C. albicans* the tested hybrid quinoline–imidazole/benzimidazole derivatives are inactive. The SAR correlation related to antibacterial activity reveals interesting data, the presence of a benzimidazole moiety being favourable for activity, the length of alkyl chain linker has no influence, while the presence of isosteres fluoro (–F), chloro (–Cl), methyl (–CH_3_) or trifloromethyl (–CF_3_) anchored in *para (4-)* position of the benzoyl moiety increase substantially the activity. We believe that all these excellent assets related to anticancer and antibacterial activity, make from our hybrid quinoline–imidazole/benzimidazole compounds bearing a phenyl group (R_2_ = –C_6_H_5_) in the *para (4)-*position of the benzoyl moiety a good candidate for future drug developing.

## Experimental

### Materials and measurements

Melting points were determined using an electrothermal Mel-Temp apparatus and were uncorrected. The NMR spectra were recorded on a Bruker Avance III 500 MHz spectrometer operating at 500 MHz for ^1^H and 125 MHz for ^13^C. The NMR apparatus is equipped with a 5 mm PABBO detection probe, and the program used for acquisition and processing data is TopSpin 3.2 PL5. The following abbreviations were used to designate chemical shift multiplicities: s = singlet, bs = broad singlet, d = doublet, dd = doublet of doublets, ad = apparent doublet, add = apparent doublet of doublets, t = triplet, at = apparent triplet, m = multiplet. Chemical shifts were reported in δ units (ppm) relative to the residual peaks of solvents (ref: DMSO, ^1^H: 2.50 ppm; ^13^C: 39.52 ppm or CDCl_3_, ^1^H: 7.26 ppm; ^13^C: 77.16 ppm. Coupling constants (*J*) were given in Hz. Infrared (IR) data were recorded as films on potassium bromide (KBr) pellets on a FT-IR VERTEX 70 Bruker spectrophotometer. The microanalyses were in satisfactory agreement with the calculated values: C, ± 0.15; H, ± 0.10; N, ± 0.30. Ultrasound assisted reactions were accomplished using Sonics (Sonics VCX-130, USA), with a nominal power of 130 W and a frequency of 20 kHz. For this ultrasonic reactor the titanium horn (diameter: 6 mm; length: 116 mm) was fixed firmly to the ultrasonic converter. The titanium probe tip was directly immersed in the used solvent. Thin layer chromatography (TLC) was carried out on Merck silica gel 60 F_254_ plates. Visualisation of the plates was achieved using a UV lamp (λ_max_ = 254 or 365 nm). The ultrasonic bath Elma Transsonic T310 (power 34.5 W, frequency 35 kHz) was used for solubilising the starting materials.

Compounds **2, 3, 4, 7a–k, 9, 11a–k** and **13a–k** were previous reported^21^.

### General procedure for the synthesis of starting materials 5 and 10

Imidazole (5.5 mmol)/benzimidazole (5.5 mmol) are solubilized in 20 mL anhydrous acetonitrile (ACN) and NaH 60% is gradually added (15 mmol, 0.6 g), suspended in anhydrous ACN, this being pre-washed with *n*-hexane to remove the mineral oil. To the resulting mixture was added the corresponding propionamide **3** (5 mmol), solubilized in 25 mL of anhydrous ACN. After addition of the reagents, the reaction is continued for 8 h at room temperature (TLC monitoring). Reaction processing consisted of simple filtration of the precipitate formed, and the solution in the flask was concentrated on a rotaevaporator. The precipitate obtained is purified by separation on a chromatographic column, using silica gel as a solid stationary phase, and a mixture of CH_2_Cl_2_ and CH_3_OH (96/4) as eluent.

### General procedure for the synthesis of quaternary salts 8a–k and 12a–k

The solution that contains parental hybrid compound **5** or **10** (1 mmol) and corresponding phenacyl bromide/chloride **6a–k** (1.2 mmol) was placed in a reaction vessel and was exposed under US irradiation. The best results were obtained by applying a pulse irradiation (5 s pulse/5 s pause, 100% from the full power of the generator). Once the irradiation cycle was completed, the reaction vessel was removed and the obtained precipitate was separated by filtration and was washed with 5–7 mL of acetone. No other purification was required. The reactions take place under ultrasound irradiation between 20 and 100 min.

### Spectral data of starting materials 5 and 10

**Figure 4:**
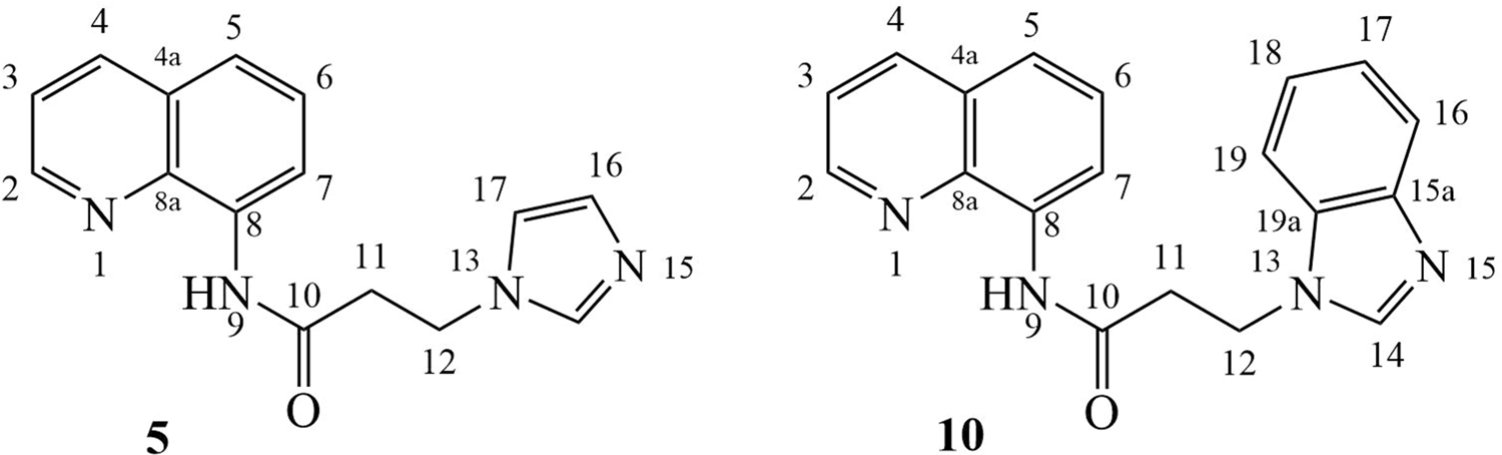
The number of atoms attributed to starting materials **5** and **10**.

Figure 4 shows the numbering of the starting compounds.

#### 3-(1H-imidazol-1-yl)-N-(quinolin-8-yl)propanamide (5)

White powder, 42% yield, m.p. = 167–170 °C. ^**1**^**H-NMR** (500 MHz, DMSO-*d6*): δ = 3.14 (t, *J* = 6.5 Hz, 2H, H_11_), 4.32 (t, *J* = 6.5 Hz, 2H, H_12_), 6.85 (s, 1H, H_16_), 7.21 (s, 1H, H_17_), 7.57 (t, *J* = 8.0 Hz, 1H, H_6_), 7.62 (dd, *J* = 4.0 Hz, *J* = 8.0 Hz, 1H, H_3_), 7.67–7.66 (m, 2H, H_5_, H_14_), 8.39 (d, *J* = 8.0 Hz, 1H, H_4_), 8.62 (d, *J* = 7.5 Hz, 1H, H_7_), 8.92 (ad, *J* = 4.0 Hz, 1H, H_2_), 10.23 (s, 1H, H_9_). ^**13**^**C-NMR** (125 MHz, DMSO-*d6*): δ = 37.8, 42.2, 117.0, 119.3, 122.1, 126.9, 127.8, 128.3, 134.3, 136.5, 137.3, 138.1, 148.8, 169.2. Anal. Calcd. for C_15_H_14_N_4_O: C, 67.65; H, 5.30; N, 21.04; found: C, 67.75; H, 5.20; N, 21.24.

#### 3-(1H-benzo[sd]imidazol-1-yl)-N-(quinolin-8-yl)propanamide (10)

White powder, 54% yield, m.p. = 160–163 °C. ^**1**^**H-NMR** (500 MHz, CDCl_3_): δ = 3.12 (t, *J* = 6.5 Hz, 2H, H_11_), 4.70 (t, *J* = 6.5 Hz, 2H, H_12_), 7.34–7.27 (m, 2H, H_6_, H_18_), 7.42 (dd, *J* = 4.5 Hz, *J* = 8.5 Hz, 1H, H_3_), 7.55–7.49 (m, 3H, H_5_, H_17_, H_19_), 7.79 (d, *J* = 8.0 Hz, 1H, H_7_), 8.07 (s, 1H, H_14_), 8.13 (add, *J* = 8.5 Hz, 1H, H_4_), 8.73–8.70 (m, 2H, H_2_, H_16_), 9.73 (bs, 1H, H_9_). ^**13**^**C-NMR** (125 MHz, CDCl_3_): δ = 37.6, 40.5, 109.5, 116.8, 120.6, 121.8, 122.1, 122.3, 123.1, 127.4, 128.0, 133.6, 133.9, 136.5, 138.2, 143.7, 144.0, 148.3, 167.9. Anal. Calcd. for C_19_H_16_N_4_O: C, 72.13; H, 5.10; N, 17.71; found: C, 72.03; H, 5.15; N, 17.61.

### Spectral data of quaternary salts 8a–k and 12a–k

**Figure 5:**
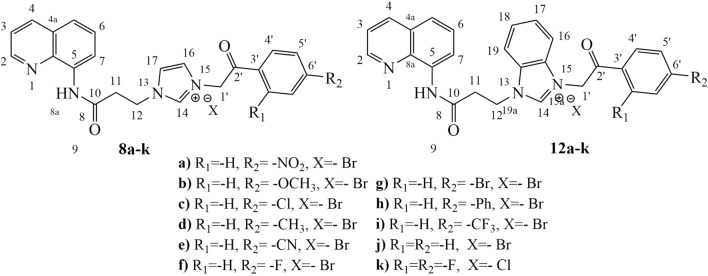
The number of atoms attributed to quaternary salts **8a–k** and **12a–k**.

Figure 5 shows the numbering of the quaternary salts.

#### 3-(2-(4-Nitrophenyl)-2-oxoethyl)-1-(3-oxo-3-(quinolin-8-ylamino)propyl)-1H-imidazol-3-ium bromide (8a)

Yellow powder, 80% yield; m.p. = 190–192 °C. ^**1**^**H-NMR** (500 MHz, DMSO-*d6*): δ = 3.34 (at, *J* = 6.5 Hz, 2H, H_11_), 4.65 (t, *J* = 6.5 Hz, 2H, H_12_), 6.13 (s, 2H, H_1’_), 7.58 (t, *J* = 8.0 Hz, 1H, H_6_), 7.65 (dd, *J* = 4.0 Hz, *J* = 8.0 Hz, 1H, H_3_), 7.72–7.69 (m, 2H, H_5_, H_16_), 7.94 (s, 1H, H_17_), 8.26 (d, *J* = 9.0 Hz, 2H, 2xH_4’_), 8.44–8.41 (m, 3H, H_4_, 2xH_5’_), 8.60 (d, *J* = 8.0 Hz, 1H, H_7_), 8.95 (add, *J* = 4.0 Hz, 1H, H_2_), 9.20 (s, 1H, H_14_), 10.37 (bs, 1H, H_9_). ^**13**^**C-NMR** (125 MHz, DMSO-*d6*): δ = 36.2, 45.3, 55.8, 117.3, 122.2, 122.3, 122.4, 123.9, 124.1, 126.8, 127.9, 129.6, 134.2, 136.6, 137.7, 138.2, 138.3, 148.9, 150.5, 168.6, 190.7. **IR** (KBr) v/cm^-1^: 3327, 3014, 2976, 1715, 1684, 1524, 1343, 1182. Anal. Calcd. for C_23_H_20_BrN_5_O_4_: C, 54.13; H, 3.95; N, 13.72; found: C, 54.18; H, 3.90; N, 13.62.

#### 3-(2-(4-Methoxyphenyl)-2-oxoethyl)-1-(3-oxo-3-(quinolin-8-ylamino)propyl)-1H-imidazol-3-ium bromide (8b)

White powder, 87% yield; m.p. = 204–205 °C. ^**1**^**H-NMR** (500 MHz, DMSO-*d6*): δ = 3.34 (at, *J* = 7.0 Hz, 2H, H_11_), 3.89 (s, 3H, *p*-OCH_3_, 4.64 (t, *J* = 7.0 Hz, 2H, H_12_), 6.10 (s, 2H, H_1’_), 7.14 (d, *J* = 9.0 Hz, 2H, 2xH_5’_), 7.58 (t, *J* = 8.0 Hz, 1H, H_6_), 7.65 (dd, *J* = 4.0 Hz, *J* = 8.0 Hz, 1H, H_3_), 7.71–7.69 (m, 2H, H_5_, H_17_), 7.91 (s, 1H, H_16_), 8.01 (d, *J* = 9.0 Hz, 2H: 2xH_4’_), 8.42 (add, *J* = 8.0 Hz, 1H, H_4_), 8.59 (d, *J* = 8.0 Hz, 1H, H_7_), 8.95 (add, *J* = 4.0 Hz, 1H, H_2_), 9.20 (s, 1H, H_14_), 10.37 (s, 1H, H_9_). ^**13**^**C-NMR** (125 MHz, DMSO-*d6*): δ = 36.2, 45.2, 55.0, 55.7 (*p*-OCH_3_), 114.3, 117.3, 122.1, 122.2, 122.3, 124.0, 126.5, 126.8, 127.9, 130.5, 134.2, 136.6, 137.7, 138.2, 148.9, 164.0, 168.6, 189.5. **IR** (KBr) v/cm^−1^: 3325, 3018, 2960, 1678, 1603, 1560, 1230, 1162. Anal. Calcd. for C_24_H_23_BrN_4_O_3_: C, 58.19; H, 4.68; N, 11.31; found: C, 58.29; H, 4.78; N, 11.11.

#### 3-(2-(4-Chlorophenyl)-2-oxoethyl)-1-(3-oxo-3-(quinolin-8-ylamino)propyl)-1H-imidazol-3-ium bromide (8c)

Beige powder, 83% yield; m.p. = 218–220 °C. ^**1**^**H-NMR** (500 MHz, DMSO-*d6*): δ = 3.35 (at, *J* = 6.5 Hz, 2H, H_11_), 4.65 (t, *J* = 6.5 Hz, 2H, H_12_), 6.09 (s, 2H, H_1’_), 7.58 (t, *J* = 8.0 Hz, 1H, H_6_), 7.64 (dd, *J* = 4.0 Hz, *J* = 8.0 Hz, 1H, H_3_), 7.73–7.69 (m, 4H, H_5_, 2xH_5’_, H_16_), 7.94 (s, 1H, H_17_), 8.05 (d, *J* = 9.0 Hz, 2H, 2xH_4’_), 8.42 (add, *J* = 8.0 Hz, 1H, H_4_), 8.59 (d, *J* = 8.0 Hz, 1H, H_7_), 8.95 (add, *J* = 4.0 Hz, 1H, H_2_), 9.22 (s, 1H, H_14_), 10.36 (s, 1H, H_9_). ^**13**^**C-NMR** (125 MHz, DMSO-*d6*): δ = 36.2, 45.2, 55.4, 117.3, 122.1, 122.3, 122.4, 124.0, 126.8, 127.9, 129.2, 130.0, 132.4, 134.2, 136.6, 137.7, 138.2, 139.3, 148.9, 168.6, 190.5. **IR** (KBr) v/cm^-1^: 3332, 3008, 2987, 1670, 1530, 732. Anal. Calcd. for C_23_H_20_BrClN_4_O_2_: C, 55.27; H, 4.03; N, 11.21; found: C, 55.37; H, 4.08; N, 11.06.

#### 3-(2-Oxo-2-(p-tolyl)ethyl)-1-(3-oxo-3-(quinolin-8-ylamino)propyl)-1H-imidazol-3-ium bromide (8d)

White powder, 90% yield; m.p. = 195–198 °C. ^**1**^**H-NMR** (500 MHz, DMSO-*d6*): δ = 2.41 (s, 3H, *p*-CH_3_), 3.34 (at, *J* = 6.0 Hz, 2H, H_11_), 4.64 (t, *J* = 6.0 Hz, 2H, H_12_), 6.04 (s, 2H, H_1’_), 7.43 (d, *J* = 8.5 Hz, 2H, 2xH_5’_), 7.58 (t, *J* = 8.0 Hz, 1H, H_6_), 7.65 (dd, *J* = 4.0 Hz, *J* = 8.0 Hz, 1H, H_3_), 7.70 (d, *J* = 8.0 Hz, 1H, H_5_), 7.73 (bs, 1H, H_16_), 7.93 (ad, *J* = 8.5 Hz, 3H, 2xH_4’_, H_17_), 8.42 (add, *J* = 8.0 Hz, 1H, H_4_), 8.60 (d, *J* = 8.0 Hz, 1H, H_7_), 8.95 (add, *J* = 4.0 Hz, 1H, H_2_), 9.21 (s, 1H, H_14_), 10.37 (s, 1H, H_9_). ^**13**^**C-NMR** (125 MHz, DMSO-*d6*): δ = 21.3 (*p*-CH_3_), 36.2 (C_11_), 45.2 (C_12_), 55.2 (C_1’_), 117.3 (C_7_), 122.1 (C_3_), 122.2 (C_5_), 122.3 (C_17_), 124.0 (C_16_), 126.8 (C_6_), 127.9 (C_4a_), 128.2 (2xC_4’_), 129.6 (2xC_5’_), 131.2 (C_3’_), 134.2 (C_8_), 136.6 (C_4_), 137.7 (C_14_), 138.2 (C_8a_), 145.1 (C_6’_), 148.9 (C_2_), 168.6 (C_10_), 190.7 (C_2’_). **IR** (KBr) v/cm^-1^: 3262, 3035, 2993, 1745, 1686, 1541, 1238, 1184, 1074. Anal. Calcd. for C_24_H_23_BrN_4_O_2_: C, 60.13; H, 4.84; N, 11.69; found: C, 60.03; H, 4.74; N, 11.79.

#### 3-(2-(4-Cyanophenyl)-2-oxoethyl)-1-(3-oxo-3-(quinolin-8-ylamino)propyl)-1H-imidazol-3-ium bromide (8e)

White powder, 80% yield; m.p. = 213–216 °C. ^**1**^**H-NMR** (500 MHz, DMSO-*d6*): δ = 3.34 (2H: H_11_, *J* = 6.0 Hz, at), 4.65 (2H: H_12_, *J* = 6.0 Hz, t), 6.11 (2H: H_1’_, s), 7.59 (1H: H_6_, *J* = 8.0 Hz, t), 7.65 (1H: H_3_, *J* = 4.0 Hz, *J* = 8.0 Hz, dd), 7.67 (1H: H_5_, *J* = 8.0 Hz, d), 7.72 (1H: H_16,_ bs), 7.94 (1H: H_17,_ bs), 8.12 (2H: 2xH_5’_, *J* = 8.0 Hz, d), 8.18 (2H: 2xH_4’_, *J* = 8.0 Hz, d), 8.42 (1H: H_4_, *J* = 8.0 Hz, d), 8.59 (1H: H_7_, *J* = 8.0 Hz, d), 8.95 (1H: H_2_, *J* = 4.0 Hz, ad), 9.20 (1H: H_14_, s), 10.37 (1H: H_9_, s). ^**13**^**C-NMR** (125 MHz, DMSO-*d6*): δ = 36.2, 45.2, 55.7, 116.1, 117.3, 117.9, 122.1, 122.3, 122.3, 123.9, 126.8, 127.9, 128.7, 133.0, 134.2, 136.6, 136.9, 137.7, 138.2, 148.9, 168.6, 190.9. **IR** (KBr) v/cm^-1^: 3325, 3026, 2973, 2234, 1725, 1530, 1230, 1078. Anal. Calcd. for C_24_H_20_BrN_5_O_2_: C, 58.79; H, 4.11; N, 14.28; found: C, 58.89; H, 4.01; N, 14.08.

#### 3-(2-(4-Fluorophenyl)-2-oxoethyl)-1-(3-oxo-3-(quinolin-8-ylamino)propyl)-1H-imidazol-3-ium bromide (8f)

White powder, 83% yield; m.p. = 206–209 °C. ^**1**^**H-NMR** (500 MHz, DMSO-*d6*): δ = 3.35 (at, *J* = 6.5 Hz, 2H, H_11_), 4.65 (t, *J* = 6.5 Hz, 2H, H_12_), 6.08 (s, 2H, H_1’_), 7.47 (t, *J* = 8.5 Hz, 2H, 2xH_5’_), 7.58 (t, *J* = 8.0 Hz, 1H, H_6_), 7.65 (dd, *J* = 4.0 Hz, *J* = 8.0 Hz, 1H, H_3_), 7.70 (d, *J* = 8.0 Hz, 1H, H_5_), 7.73 (bs, 1H, H_16_), 7.93 (bs, 1H, H_17_), 8.13 (aq, *J* = 8.5 Hz, 2H, 2xH_4’_), 8.42 (add, *J* = 8.0 Hz, 1H, H_4_), 8.59 (d, *J* = 8.0 Hz, 1H, H_7_), 8.95 (ad, *J* = 4.0 Hz, 1H, H_2_), 9.22 (s, 1H, H_14_), 10.37 (s, 1H, H_9_). ^**13**^**C-NMR** (125 MHz, DMSO-*d6*): δ = 36.2, 45.2, 55.3, 116.2, 117.3, 122.1, 122.3, 122.4, 124.0, 126.8, 127.9, 130.5, 131.2, 134.2, 136.6, 137.7, 138.2, 148.9, 165.6, 168.6, 190.0. **IR** (KBr) v/cm^−1^: 3295, 3024, 2975, 1701, 1692, 1543, 1233, 1174. Anal. Calcd. for C_23_H_20_BrFN_4_O_2_: C, 57.15; H, 4.17; N, 11.59; found: C, 57.05; H, 4.12; N, 11.74.

#### 3-(2-(4-Bromophenyl)-2-oxoethyl)-1-(3-oxo-3-(quinolin-8-ylamino)propyl)-1H-imidazol-3-ium bromide (8g)

White powder, 85% yield; m.p. = 189–192 °C. ^**1**^**H-NMR** (500 MHz, DMSO-*d6*): δ = 3.35 (at, *J* = 6.5 Hz, 2H, H_11_), 4.64 (t, *J* = 6.5 Hz, 2H, H_12_), 6.04 (s, 2H, H_1’_), 7.58 (t, *J* = 8.0 Hz,1H, H_6_), 7.65 (dd, *J* = 4.5 Hz, *J* = 8.5 Hz, 1H, H_3_), 7.70–7.69 (m, 2H, H_5_, H_16_), 7.86 (d, *J* = 8.5 Hz, 2H, 2xH_5’_), 7.92 (bs, 1H, H_17_), 7.96 (d, *J* = 8.5 Hz, 2H, 2xH_4’_), 8.42 (d, *J* = 8.5 Hz, 1H, H_4_), 8.59 (d, *J* = 8.0 Hz, 1H, H_7_), 8.95 (ad, *J* = 4.5 Hz, 1H, H_2_), 9.18 (s, 1H, H_14_), 10.37 (s, 1H, H_9_). ^**13**^**C-NMR** (125 MHz, DMSO-*d6*): δ = 36.2, 45.2, 55.3, 117.3, 122.2, 122.3, 122.4, 124.0, 126.8, 127.9, 128.6, 130.1, 132.2, 132.7, 134.2, 136.6, 137.7, 138.2, 148.9, 168.6, 190.7. **IR** (KBr) v/cm^−1^: 3241, 3027, 2986, 1742, 1702, 1529, 1223, 1175, 681. Anal. Calcd. for C_23_H_20_Br_2_N_4_O_2_: C, 50.76; H, 3.70; N, 10.29; found: C, 50.66; H, 3.65; N, 10.44.

#### 3-(2-([1,1′-Biphenyl]-4-yl)-2-oxoethyl)-1-(3-oxo-3-(quinolin-8-ylamino)propyl)-1H-imidazol-3-ium bromide (8h)

White powder, 86% yield; m.p. = 211–214 °C. ^**1**^**H-NMR** (500 MHz, DMSO-*d6*): δ = 3.36 (at, *J* = 6.5 Hz, 2H, H_11_), 4.66 (t, *J* = 6.5 Hz, 2H, H_12_), 6.11 (s, 2H, H_1’_), 7.46 (t, *J* = 7.5 Hz, 1H, H_10’_), 7.53 (t, *J* = 7.5 Hz, 2H, 2xH_9’_), 7.59 (t, *J* = 8.0 Hz, 1H, H_6_), 7.65 (dd, *J* = 4.0 Hz, *J* = 8.0 Hz, 1H, H_3_) 7.70 (d, *J* = 8.0 Hz, 1H, H_5_), 7.74 (bs, 1H, H_16_), 7.80 (d, *J* = 7.5 Hz, 2H, 2xH_8’_), 7.94 (ad, *J* = 8.5 Hz, 3H, 2xH_5’_, H_17_), 8.12 (d, *J* = 8.5 Hz, 2H, 2xH_4’_), 8.43 (d, *J* = 8.0 Hz, 1H, H_4_), 8.61 (d, *J* = 8.0 Hz, 1H, H_7_), 8.95 (ad, *J* = 4.0 Hz, 1H, H_2_), 9.23 (s, 1H, H_14_), 10.38 (s, 1H, H_9_). ^**13**^**C-NMR** (125 MHz, DMSO-*d6*): δ = 36.2, 45.2, 55.4, 117.3, 122.2, 122.3, 122.4, 124.0, 126.8, 127.1, 127.1, 127.9, 128.7, 128.9, 129.1, 132.5, 134.2, 136.6, 137.8, 138.2, 138.5, 145.7, 148.9, 168.6, 190.8. **IR** (KBr) v/cm^−1^: 3238, 3015, 2986, 1698, 1680, 1575, 1215. Anal. Calcd. for C_29_H_25_BrN_4_O_2_: C, 64.33; H, 4.65; N, 10.35; found: C, 64.43; H, 4.60; N, 10.25.

#### 3-(2-Oxo-2-(4-(trifluoromethyl)phenyl)ethyl)-1-(3-oxo-3-(quinolin-8-ylamino)propyl)-1H-imidazol-3-ium bromide (8i)

White powder, 79% yield; m.p. = 219–221 °C. ^**1**^**H-NMR** (500 MHz, DMSO-*d6*): δ = 3.35 (at, *J* = 6.5 Hz, 2H, H_11_), 4.65 (t, *J* = 6.5 Hz, 2H, H_12_), 6.11 (s, 2H, H_1’_), 7.59 (t, *J* = 8.0 Hz, 1H, H_6_), 7.65 (dd, *J* = 4.5 Hz, *J* = 8.5 Hz, 1H, H_3_), 7.71–7.69 (m, 2H, H_5_, H_16_), 7.93 (bs, 1H, H_17_), 8.02 (d, *J* = 8.5 Hz, 2H, 2xH_5’_), 8.23 (d, *J* = 8.5 Hz, 2H, 2xH_4’_), 8.42 (add, *J* = 8.5 Hz, 1H, H_4_,), 8.60 (d, *J* = 8.0 Hz, 1H, H_7_), 8.95 (add, *J* = 4.5 Hz, 1H, H_2_), 9.19 (s, 1H, H_14_), 10.38 (s, 1H, H_9_,). ^**13**^**C-NMR** (125 MHz, DMSO-*d6*): δ = 36.2, 45.3, 55.6, 117.3, 122.2, 122.3, 122.4, 123.6, 124.0, 126.1, 126.9, 127.9, 129.0, 133.5, 134.2, 136.6, 136.9, 137.7, 138.2, 148.9, 168.6, 191.0. **IR** (KBr) v/cm^−1^: 3312, 3021, 2983, 1680, 1586, 1238, 1165. Anal. Calcd. for C_24_H_20_BrF_3_N_4_O_2_: C, 54.05; H, 3.78; N, 10.50; found: C, 54.15; H, 3.73; N, 10.40.

#### 3-(2-Oxo-2-phenylethyl)-1-(3-oxo-3-(quinolin-8-ylamino)propyl)-1H-imidazol-3-ium bromide (8j)

White powder, 82% yield; m.p. = 197–201 °C. ^**1**^**H-NMR** (500 MHz, DMSO-*d6*): δ = 3.35 (at, *J* = 6.5 Hz, 2H, H_11_), 4.65 (t, *J* = 6.5 Hz, 2H, H_12_), 6.08 (s, 2H, H_1’_), 7.76–7.57 (m, 7H, H_3_, H_5_, H_6_, 2xH_5’_, H_6’_, H_16_), 7.93 (bs, 1H, H_17_), 8.04 (d, *J* = 7.0 Hz, 2H, 2xH_4’_), 8.42 (add, *J* = 8.5 Hz, 1H, H_4_), 8.60 (d, *J* = 8.0 Hz, 1H, H_7_), 8.95 (add, *J* = 4.0 Hz, 1H, H_2_), 9.21 (s, 1H, H_14_), 10.38 (s, 1H, H_9_). ^**13**^**C-NMR** (125 MHz, DMSO-*d6*): δ = 36.2, 45.2, 55.4, 117.3, 122.2, 122.3, 122.4, 124.0, 126.8, 127.9, 128.1, 129.1, 133.6, 134.2, 134.5, 136.6, 137.7, 138.2, 148.9, 168.6, 191.3. **IR** (KBr) v/cm^-1^: 3321, 3019, 2969, 1698, 1575, 1116. Anal. Calcd. for C_23_H_21_BrN_4_O_2_: C, 59.36; H, 4.55; N, 12.04; found: C, 59.46; H, 4.50; N, 12.14.

#### 3-(2-(2,4-Difluorophenyl)-2-oxoethyl)-1-(3-oxo-3-(quinolin-8-ylamino)propyl)-1H-imidazol-3-ium chloride (8k)

White powder, 76% yield, m.p. = 221–223 °C. ^**1**^**H-NMR** (500 MHz, DMSO-*d6*): δ = 3.34 (at, *J* = 6.5 Hz, 2H, H_11_), 4.64 (t, *J* = 6.5 Hz, 2H, H_12_), 5.90 (s, 2H, H_1’_), 7.35 (td, *J* = 8.5 Hz, 2H, 2xH_5’_), 7.61–7.56 (m, 2H, H_7’_, H_6_), 7.65 (dd, *J* = 4.5 Hz, *J* = 8.5 Hz,1H, H_3_,), 7.70–7.69 (m, 2H, H_5_, H_17_), 7.92 (bs, 1H, H_17_), 8.05 (q, *J* = 8.5 Hz, 2H, 2xH_4’_), 8.42 (add, *J* = 8.5 Hz, 1H, H_4_), 8.59 (d, *J* = 7.5 Hz, 1H, H_7_), 8.94 (add, *J* = 4.5 Hz, 1H, H_2_), 9.21 (s, 1H, H_14_), 10.38 (s, 1H, H_9_). ^**13**^**C-NMR** (125 MHz, DMSO-*d6*): δ = 36.2, 45.2, 57.8, 105.5, 113.1, 117.3, 119.1, 122.2, 122.2, 122.3, 124.0, 126.8, 127.9, 132.7, 134.2, 136.6, 137.8, 138.2, 148.9, 162.5, 165.9, 168.6, 187.9. **IR** (KBr) v/cm^−1^: 3319, 3006, 2974, 1715, 1685, 1540, 1221, 1123. Anal. Calcd. for C_23_H_19_ClF_2_N_4_O_2_: C, 60.47; H, 4.19; N, 12.26; found: C, 60.57; H, 4.09; N, 12.16.

#### 3-(2-(4-Nitrophenyl)-2-oxoethyl)-1-(3-oxo-3-(quinolin-8-ylamino)propyl)-1H-benzo[d]imidazol-3-ium bromide (12a)

Yellowish powder, 76% yield, m.p. = 165–168 °C. ^**1**^**H-NMR** (500 MHz, DMSO-*d6*): δ = 3.44 (at, *J* = 6.5 Hz, 2H, H_11_), 4.99 (t, *J* = 6.5 Hz, 2H, H_12_), 6.49 (s, 2H, H_1’_), 7.57 (t, *J* = 8.0 Hz, 1H, H_6_) 7.63 (dd, *J* = 4.5 Hz, *J* = 8.5 Hz,1H, H_3_), 7.70–7.67 (m, 2H, H_5_, H_18_), 7.75 (t, *J* = 8.5 Hz, 1H, H_17_), 8.09 (d, *J* = 8.5 Hz, 1H, H_19_), 8.25 (d, *J* = 8.5 Hz, 1H, H_16_), 8.34 (d, *J* = 8.5 Hz, 2H, 2xH_4’_), 8.40 (d, *J* = 8.5 Hz, 1H, H_4_), 8.46 (d, *J* = 8.5 Hz, 2H, 2xH_5’_), 8.57 (d, *J* = 8.0 Hz, 1H, H_7_), 8.91 (ad, *J* = 4.5 Hz, 1H, H_2_), 9.79 (s, 1H, H_14_), 10.38 (s, 1H, H_9_). ^**13**^**C-NMR** (125 MHz, DMSO-*d6*): δ = 35.2, 43.2, 53.7, 114.0, 114.1, 117.4, 122.2, 122.4, 124.1, 126.7, 126.8, 126.9, 127.9, 129.9, 130.7, 131.9, 134.2, 136.6, 138.3, 138.4, 143.9, 148.9, 150.6, 168.8, 190.6. **IR** (KBr) v/cm^−1^: 3334, 3013, 2981, 1712, 1675, 1547, 1339. Anal. Calcd. for C_27_H_22_BrN_5_O_4_: C, 57.87; H, 3.96; N, 12.50; found: C, 57.77; H, 3.91; N, 12.65.

#### 3-(2-(4-Methoxyphenyl)-2-oxoethyl)-1-(3-oxo-3-(quinolin-8-ylamino)propyl)-1H-benzo[d]imidazol-3-ium bromide (12b)

White powder, 85% yield; m.p. = 220–221 °C. ^**1**^**H-NMR** (500 MHz, DMSO-*d6*): δ = 3.44 (at, *J* = 6.5 Hz, 2H, H_11_), 3.89 (s, 3H, *p-*OCH_3_), 4.97 (t, *J* = 6.5 Hz, 2H, H_12_), 6.36 (s, 2H, H_1’_), 7.17 (d, *J* = 9.0 Hz, 2H, 2xH_5’_), 7.58 (t, *J* = 8.0 Hz, 1H, H_6_), 7.69–7.63 (m, 3H, H_3,_ H_5_, H_18_), 7.73 (t, *J* = 8.5 Hz, 1H, H_17_), 8.02 (d, *J* = 8.0 Hz, 1H, H_19_), 8.07 (d, *J* = 9.0 Hz, 2H, 2xH_4’_), 8.23 (d, *J* = 8.5 Hz, 1H, H_16_), 8.40 (add, *J* = 8.5 Hz, 1H, H_4_), 8.58 (d, *J* = 8.0 Hz, 1H, H_7_), 8.91 (add, *J* = 4.0 Hz, 1H, H_2_), 9.79 (s, 1H, H_14_), 10.38 (s, 1H, H_9_). ^**13**^**C-NMR** (125 MHz, DMSO-*d6*): δ = 35.2, 43.2, 52.8, 55.8, 113.9, 114.0, 114.3, 117.4, 122.2, 122.4, 126.6_,_ 126.7, 126.8, 127.9, 130.7, 130.9, 131.9, 134.2, 136.6, 138.2, 144.0, 148.9, 164.2, 168.8, 189.4. **IR** (KBr) v/cm^−1^: 3247, 3002, 2986, 1675, 1601, 1548, 1243, 1165. Anal. Calcd. for C_28_H_25_BrN_4_O_3_: C, 61.66; H, 4.62; N, 10.27; found: C, 61.56; H, 4.60; N, 10.38.

#### 3-(2-(4-Chlorophenyl)-2-oxoethyl)-1-(3-oxo-3-(quinolin-8-ylamino)propyl)-1H-benzo[d]imidazol-3-ium bromide (12c)

White powder, 86% yield; m.p. = 229–230 °C. ^**1**^**H-NMR** (500 MHz, DMSO-*d6*): δ = 3.44 (at, *J* = 6.0 Hz, 2H, H_11_), 4.98 (t, *J* = 6.0 Hz, 2H, H_12_), 6.42 (s, 2H, H_1’_), 7.57 (t, *J* = 8.0 Hz, 1H, H_6_), 7.63 (dd, *J* = 4.0 Hz, *J* = 8.0 Hz, 1H, H_3_), 7.69–7.66 (m, 2H, H_15_, H_18_), 7.75–7.72 (m, 3H, 2xH_5’,_ H_17_), 8.05 (d, *J* = 8.5 Hz, 1H, H_19_), 8.12 (d, *J* = 8.5 Hz, 2H, 2xH_4’_), 8.24 (d, *J* = 8.5 Hz, 1H, H_16_), 8.40 (d, *J* = 8.0 Hz, 1H, H_4_), 8.57 (d, *J* = 8.0 Hz, 1H, H_7_), 8.91 (ad, *J* = 4.0 Hz, 1H, H_2_), 9.79 (s, 1H, H_14_), 10.38 (s, 1H, H_9_). ^**13**^**C-NMR** (125 MHz, DMSO-*d6*): δ = 35.2, 43.2, 53.3, 114.0_,_ 117.4, 122.2, 122.4, 126.6, 126.8, 126.8, 127.9, 129.2, 130.3, 130.7, 131.9, 132.5_,_ 134.2, 136.6, 138.2, 139.4, 143.9, 148.9, 168.8, 190.4. **IR** (KBr) v/cm^−1^: 3217, 3009, 2912, 1687, 1535, 739. Anal. Calcd. for C_27_H_22_BrClN_4_O_2_: C, 58.98; H, 4.03; N, 10.19; found: C, 58.88; H, 4.08; N, 10.09.

#### 3-(2-Oxo-2-(p-tolyl)ethyl)-1-(3-oxo-3-(quinolin-8-ylamino)propyl)-1H-benzo[d]imidazol-3-ium bromide (12d)

White powder, 82% yield; m.p. = 225–227 °C. ^**1**^**H-NMR** (500 MHz, DMSO-*d6*): δ = 2.43 (s, 3H, *p*-CH_3_), 3.43 (at, *J* = 6.5 Hz, 2H, H_11_), 4.97 (t, *J* = 6.5 Hz, 2H, H_12_), 6.39 (s, 2H, H_1’_), 7.46 (d, *J* = 8.0 Hz, 2H, 2xH_5’_), 7.57 (t, *J* = 8.0 Hz, 1H, H_6_), 7.63 (dd, *J* = 4.0 Hz, *J* = 8.0 Hz, 1H, H_3_), 7.69–7.65 (m, 2H, H_15_, H_18_), 7.73 (t, *J* = 8.0 Hz, 1H, H_17_), 8.03–7.99 (m, 3H, 2xH_4’_, H_19_), 8.23 (d, *J* = 8.0 Hz, 1H, H_16_), 8.40 (d, *J* = 8.0 Hz, 1H, H_4_), 8.57 (d, *J* = 8.0 Hz, 1H, H_7_), 8.91 (ad, *J* = 4.0 Hz, 1H, H_2_), 9.79 (s, 1H, H_14_), 10.38 (s, 1H, H_9_). ^**13**^**C-NMR** (125 MHz, DMSO-*d6*): δ = 21.4_,_ 35.2, 43.2, 53.3, 113.9, 114.0_,_ 117.4, 122.2, 122.4, 126.6_,_ 126.7, 126.8, 127.9, 128.5, 129.6, 130.7_,_ 131.3, 131.9, 134.3, 136.6, 138.3, 144.0, 145.3, 148.9, 168.8, 190.7. **IR** (KBr) v/cm^−1^: 3284, 3035, 2971, 1696, 1612, 1574, 1213. Anal. Calcd. for C_28_H_25_BrN_4_O_2_: C, 63.52; H, 4.76; N, 10.58; found: C, 63.42; H, 4.66; N, 10.78.

#### 3-(2-(4-Cyanophenyl)-2-oxoethyl)-1-(3-oxo-3-(quinolin-8-ylamino)propyl)-1H-benzo[d]imidazol-3-ium bromide (12e)

White powder, 90% yield; m.p. = 213–215 °C. ^**1**^**H-NMR** (500 MHz, DMSO-*d6*): δ = 3.46 (t, *J* = 6.5 Hz, 2H, H_11_), 4.99 (t, *J* = 6.5 Hz, 2H, H_12_), 6.47 (s, 2H, H_1’_), 7.57 (t, *J* = 7.5 Hz, 1H, H_6_), 7.63 (dd, *J* = 8.0 Hz, *J* = 4.0 Hz, 1H, H_3_), 7.69–7.66 (m, 2H, H_15_, H_18_), 7.75 (t, *J* = 7.5 Hz, 1H, H_17_), 8.09 (d, *J* = 8.0 Hz, 1H, H_19_), 8.16 (d, *J* = 8.5 Hz, 2H, 2xH_5’_), 8.26–8.24 (m, 2xH_4’_, 3H, H_16_), 8.40 (add, *J* = 8.0 Hz, 1H, H_4_), 8.58 (d, *J* = 7.5 Hz, 1H, H_7_), 8.92 (add, *J* = 4.0 Hz, 1H:, H_2_), 9.78 (s, 1H, H_14_), 10.38 (s, 1H, H_9_). ^**13**^**C-NMR** (125 MHz, DMSO-*d6*): δ = 35.2, 43.2, 53.5, 114.0, 116.1, 117.3, 118.0, 122.1, 122.3, 126.6_,_ 126.7, 126.8, 127.8, 129.0, 130.7, 131.8, 133.0, 134.2, 136.6, 137.0_,_ 138.2, 143.9, 148.9, 168.8, 190.8. **IR** (KBr) v/cm^−1^: 3312, 3005, 2943, 2230, 1716, 1535, 1227, 1073. Anal. Calcd. for C_28_H_22_BrN_5_O_2_: C, 62.23; H, 4.10; N, 12.96; found: C, 62.13; H, 4.15; N, 12.90.

#### 3-(2-(4-Fluorophenyl)-2-oxoethyl)-1-(3-oxo-3-(quinolin-8-ylamino)propyl)-1H-benzo[d]imidazol-3-ium bromide (12f)

White powder, 81% yield; m.p. = 230–233 °C. ^**1**^**H-NMR** (500 MHz, DMSO-*d6*): δ = 3.46 (bs, 2H, H_11_), 4.99 (bs, 2H, H_12_), 6.44 (s, 2H, H_1’_), 7.74–7.51 (m, 7H, H_3_, H_5_, H_6_, 2xH_5’_, H_17_, H_18_), 8.07 (d, *J* = 7.5 Hz, 1H, H_19_), 8.25–8.20 (m, 3H, 2xH_4’_, H_16_), 8.41 (ad, *J* = 7.5 Hz, 1H, H_4_,), 8.58 (ad, 1H, *J* = 6.0 Hz, H_7_), 8.91 (abs, 1H, H_2_), 9.81 (s, 1H, H_14_), 10.39 (s, 1H, H_9_). ^**13**^**C-NMR** (125 MHz, DMSO-*d6*): δ = 35.2, 43.2, 53.2, 114.0, 116.2, 117.3, 122.1, 122.3, 126.5_,_ 126.7, 126.8, 127.8, 130.5_,_ 130.7, 131.6, 131.9, 134.2, 136.5, 138.2, 143.9, 148.9, 165.7, 168.7, 189.9. **IR** (KBr) v/cm^−1^: 3326, 3007, 2982, 1715, 1686, 1537, 1235, 1082. Anal. Calcd. for C_27_H_22_BrFN_4_O_2_: C, 60.80; H, 4.16; N, 10.50; found: C, 60.90; H, 4.11; N, 10.40.

#### 3-(2-(4-Bromophenyl)-2-oxoethyl)-1-(3-oxo-3-(quinolin-8-ylamino)propyl)-1H-benzo[d]imidazol-3-ium bromide (12g)

White powder, 83% yield; m.p. = 213–215 °C. ^**1**^**H-NMR** (500 MHz, DMSO-*d6*): δ = 3.45 (at, *J* = 6.0 Hz, 2H, H_11_), 4.98 (at, *J* = 6.5 Hz, 2H, H_12_), 6.41 (s, 2H, H_1’_), 7.57 (t, *J* = 8.0 Hz, 1H, H_6_), 7.63 (dd, *J* = 4.0 Hz, *J* = 8.0 Hz, 1H, H_3_), 7.69–7.66 (m, 2H, H_5_, H_18_), 7.74 (t, *J* = 8.0 Hz, 1H, H_17_), 7.89 (d, *J* = 8.5 Hz, 2H, 2xH_5’_), 8.07–8.02 (d, 3H, 2xH_4’_, H_19_), 8.24 (d, *J* = 8.0 Hz, 1H, H_16_), 8.41 (add, *J* = 8.0 Hz, 1H, H_4_), 8.58 (d, *J* = 8.0 Hz, 1H, H_7_), 8.92 (add, *J* = 4.0 Hz, 1H, H_2_), 9.78 (s, 1H, H_14_), 10.38 (s, 1H, H_9_). ^**13**^**C-NMR** (125 MHz, DMSO-*d6*): δ = 35.1, 43.2, 53.2, 114.0_,_ 117.3, 122.1, 122.3, 126.0_,_ 126.7, 126.8, 127.8, 128.7, 130.3, 130.7, 131.8, 130.1, 132.8_,_ 134.2, 136.6, 138.2, 143.9, 148.9, 168.7, 190.6. **IR** (KBr) v/cm^−1^: 3300, 3020, 2970, 1732, 1700, 1530, 1229, 1170, 670. Anal. Calcd. for C_27_H_22_Br_2_N_4_O_2_: C, 54.57; H, 3.73; N, 9.43; found: C, 54.47; H, 3.63; N, 9.63.

#### 3-(2-([1,1′-Biphenyl]-4-yl)-2-oxoethyl)-1-(3-oxo-3-(quinolin-8-ylamino)propyl)-1H-benzo[d]imidazol-3-ium bromide (12h)

White powder, 81% yield; m.p. = 201–203 °C. ^**1**^**H-NMR** (500 MHz, DMSO-*d6*): δ = 3.47 (at, *J* = 6.0 Hz, 2H, H_11_), 5.00 (at, *J* = 6.0 Hz, 2H, H_12_), 6.47 (s, 2H, H_1’_), 7.47 (at, *J* = 7.5 Hz, 1H, H_10’_), 7.59–7.53 (m, 3H, H_6_, 2xH_9’_), 7.64 (dd, *J* = 4.5 Hz, *J* = 8.5 Hz, 1H, H_3_), 7.70–7.67 (m, 2H, H_15_, H_18_), 7.75 (t, *J* = 8.0 Hz, 1H, H_17_), 7.82 (d, *J* = 7.5 Hz, 2H, 2xH_8’_), 7.98 (d, *J* = 8.5 Hz, 2H, 2xH_5’_), 8.07 (d, *J* = 8.0 Hz,1H, H_19_), 8.19 (d, *J* = 8.5 Hz, 2H, 2xH_4’_), 8.25 (d, *J* = 8.0 Hz, 1H, H_16_), 8.41 (add, *J* = 8.5 Hz, 1H, H_4_), 8.59 (d, *J* = 7.5 Hz, 1H, H_7_), 8.92 (add, *J* = 4.5 Hz, 1H, H_2_) 9.82 (s, 1H, H_14_), 10.40 (s, 1H, H_9_). ^**13**^**C-NMR** (125 MHz, DMSO-*d6*): δ = 35.2, 43.2, 53.2, 114.0_,_ 117.4, 122.1, 122.3, 126.6_,_ 126.7, 126.8, 127.1, 127.9, 128.7, 129.1, 129.2, 130.7, 131.9, 132.5_,_ 134.2, 136.6, 138.2, 138.5, 143.9, 145.7, 148.9, 168.8, 190.7. **IR** (KBr) v/cm^−1^: 3294, 3035, 2971, 1689, 1674, 1573, 1212. Anal. Calcd. for C_33_H_27_BrN_4_O_2_: C, 67.01; H, 4.60; N, 9.47; found: C, 67.11; H, 4.55; N, 9.37.

#### 3-(2-Oxo-2-(4-(trifluoromethyl)phenyl)ethyl)-1-(3-oxo-3-(quinolin-8-ylamino)propyl)-1H-benzo[d]imidazol-3-ium bromide (12i)

White powder, 74% yield; m.p. = 217–218 °C. ^**1**^**H-NMR** (500 MHz, DMSO-*d6*): δ = 3.46 (at, *J* = 6.0 Hz, 2H, H_11_), 5.00 (at, *J* = 6.5 Hz, 2H, H_12_), 6.49 (s, 2H, H_1’_), 7.57 (t, *J* = 8.0 Hz, 1H, H_6_), 7.63 (dd, *J* = 4.0 Hz, *J* = 8.0 Hz, 1H, H_3_), 7.70–7.67 (m, 2H, H_15_, H_18_), 7.75 (t, *J* = 8.0 Hz, 1H, H_17_), 8.10–8.04 (m, 3H, 2xH_5’,_ H_19_), 8.25 (d, *J* = 8.0 Hz, 1H, H_16_), 8.30 (d, *J* = 8.0 Hz, 2H, 2xH_4’_), 8.41 (add, *J* = 8.0 Hz, 1H, H_4_,), 8.58 (d, *J* = 8.0 Hz, 1H, H_7_), 8.92 (add, *J* = 4.0 Hz, 1H, H_2_), 9.80 (s, 1H, H_14_), 10.39 (s, 1H, H_9_). ^**13**^**C-NMR** (125 MHz, DMSO-*d6*): δ = 35.2, 43.2, 53.5, 114.0_,_ 117.3, 122.1, 122.3, 123.6, 126.0, 126.6, 126.7, 126.8, 127.8, 129.3, 130.7, 131.8, 133.5, 134.2, 136.6, 137.0, 138.2, 143.9, 148.9, 168.7, 190.8. **IR** (KBr) v/cm^−1^: 3342, 3019, 2975, 1720, 1643, 1523, 1241, 1117. Anal. Calcd. for C_28_H_22_BrF_3_N_4_O_2_: C, 57.65; H, 3.80; N, 9.60; found: C, 57.55; H, 3.85; N, 9.50.

#### 3-(2-Oxo-2-phenylethyl)-1-(3-oxo-3-(quinolin-8-ylamino)propyl)-1H-benzo[d]imidazol-3-ium bromide (12j)

White powder, 80% yield; m.p. = 196–198 °C. ^**1**^**H-NMR** (500 MHz, DMSO-*d6*): δ = 3.46 (at, *J* = 6.0 Hz, 2H, H_11_), 4.98 (at, *J* = 6.0 Hz, 2H, H_12_), 6.42 (s, 2H, H_1’_), 7.57 (t, *J* = 8.0 Hz, 1H, H_6_), 7.69–7.62 (m, 5H, H_3_, H_5_, 2xH_5’_, H_6’_), 7.74 (t, *J* = 8.0 Hz, 1H, H_17_), 7.79 (t, *J* = 8.0 Hz, 1H, H_18_), 8.05 (d, *J* = 8.0 Hz, 1H, H_19_), 8.11 (d, *J* = 7.5 Hz, 1H, 2xH_4’_), 8.24 (d, *J* = 8.0 Hz, 1H, H_16_), 8.41 (add, *J* = 8.0 Hz, 1H, H_4_,), 8.58 (d, *J* = 7.5 Hz, 1H, H_7_), 8.92 (add, *J* = 4.0 Hz, 1H, H_2_), 9.78 (s, 1H, H_14_), 10.39 (s, 1H, H_9_). ^**13**^**C-NMR** (125 MHz, DMSO-*d6*): δ = 35.1, 43.2, 53.2, 114.0, 117.3, 122.1, 122.3, 126.5_,_ 126.7, 126.8, 127.8, 128.4, 129.0, 130.7, 131.9, 133.7, 134.2, 134.5, 136.6, 138.2, 143.9, 148.9, 168.7, 191.1. **IR** (KBr) v/cm^−1^: 3273, 3031, 2976, 1698, 1684, 1589, 1207. Anal. Calcd. for C_27_H_23_BrN_4_O_2_: C, 62.92; H, 4.50; N, 10.87; found: C, 62.82; H, 4.45; N, 10.97.

#### 3-(2-(2,4-Difluorophenyl)-2-oxoethyl)-1-(3-oxo-3-(quinolin-8-ylamino)propyl)-1H-benzo[d]imidazol-3-ium chloride (12k)

White powder, 70% yield; m.p. = 203–205 °C. ^**1**^**H-NMR** (500 MHz, DMSO-*d6*): δ = 3.44 (at, *J* = 6.5 Hz, 2H, H_11_), 4.98 (t, *J* = 6.5 Hz, 2H, H_12_), 6.24 (s, 2H, H_1’_), 7.36 (td, *J* = 8.5 Hz, 1H, H_5’_), 7.57 (t, *J* = 7.5 Hz, 1H, H_6_), 7.64–7.60 (m, 2H, H_3_, H_7’_), 7.69–7.65 (m, 2H, H_5_, H_18_), 7.73 (t, *J* = 8.0 Hz,1H, H_17_), 8.09–8.05 (m, 2H, H_4’_, H_19_), 8.24 (d, *J* = 8.0 Hz, 1H, H_16_), 8.41 (add, *J* = 8.0 Hz, 1H, H_4_), 8.57 (d, *J* = 7.5 Hz, 1H, H_7_), 8.92 (add, *J* = 4.0 Hz, 1H, H_2_), 9.84 (s, 1H, H_14_), 10.39 (s, 1H, H_9_). ^**13**^**C-NMR** (125 MHz, DMSO-*d6*): δ = 35.2, 43.1, 55.8, 105.5, 113.0, 113.9_,_ 114.2, 117.5, 119.3, 122.1, 122.4, 126.5_,_ 126.7, 126.8, 127.9, 130.6, 131.9, 132.7_,_ 134.2, 136.6, 138.2, 143.9, 148.9, 162.8, 166.0, 168.7, 187.7. **IR** (KBr) v/cm^−1^: 3332, 3006, 2973, 1698, 1680, 1575, 1397, 1231. Anal. Calcd. for C_27_H_21_ClF_2_N_4_O_2_: C, 63.97; H, 4.18; N, 11.05; found: C, 63.87; H, 4.13; N, 11.25.

### Cell proliferation assay

The in vitro biological tests were performed to the National Cancer Institute (NCI, USA), under the Developmental Therapeutics Program (DTP). The DTP screens include the NCI 60 cell line screen and, as appropriate, the hollow fiber assay and relevant human tumor xenograft and rodent tumor models.

The operation of this screen utilizes 60 different human tumor cell lines, representing leukemia, melanoma and cancers of the lung, colon, brain, ovary, breast, prostate, and kidney. The aim is to prioritize for further evaluation^26,38^, synthetic compounds or natural product samples showing selective growth inhibition or cell killing of particular tumor cell lines. This screen is unique in that the complexity of a 60 cell line dose response produced by a given compound results in a biological response pattern which can be utilized in pattern recognition algorithms via **COMPARE** program (See: http://dtp.nci.nih.gov/docs/compare/compare.html).

The screening is beginning with the evaluation of all compounds against the 60 cell lines at a single dose of 10^–5^ M. The output from the single dose screen is reported as a mean graph and is available for analysis by the COMPARE program.

### The standard NCI/DTP methodology of the in vitro cancer screen

See: https://dtp.cancer.gov/discovery_development/nci-60/methodology.html.

The human tumor cell lines of the cancer screening panel are grown in RPMI 1640 medium containing 5% fetal bovine serum and 2 mM l-glutamine. For a typical screening experiment, cells are inoculated into 96 well microtiter plates in 100 µL at plating densities ranging from 5000 to 40,000 cells/well depending on the doubling time of individual cell lines. After cell inoculation, the microtiter plates are incubated at 37 °C, 5% CO_2_, 95% air and 100% relative humidity for 24 h prior to addition of experimental drugs.

After 24 h, two plates of each cell line are fixed in situ with TCA, to represent a measurement of the cell population for each cell line at the time of drug addition (Tz). Experimental drugs are solubilized in dimethyl sulfoxide at 400-fold the desired final maximum test concentration and stored frozen prior to use. At the time of drug addition, an aliquot of frozen concentrate is thawed and diluted to twice the desired final maximum test concentration with complete medium containing 50 µg/ml gentamicin. Additional four, tenfold or ½ log serial dilutions are made to provide a total of five drug concentrations plus control. Aliquots of 100 µL of these different drug dilutions are added to the appropriate microtiter wells already containing 100 µL of medium, resulting in the required final drug concentrations.

Following drug addition, the plates are incubated for an additional 48 h at 37 °C, 5% CO_2_, 95% air, and 100% relative humidity. For adherent cells, the assay is terminated by the addition of cold TCA. Cells are fixed in situ by the gentle addition of 50 µL of cold 50% (w/v) TCA (final concentration, 10% TCA) and incubated for 60 min at 4 °C. The supernatant is discarded, and the plates are washed five times with tap water and air dried. Sulforhodamine B (SRB) solution (100 µL) at 0.4% (w/v) in 1% acetic acid is added to each well, and plates are incubated for 10 min at room temperature. After staining, unbound dye is removed by washing five times with 1% acetic acid and the plates are air dried. Bound stain is subsequently solubilized with 10 mM trizma base, and the absorbance is read on an automated plate reader at a wavelength of 515 nm. For suspension cells, the methodology is the same except that the assay is terminated by fixing settled cells at the bottom of the wells by gently adding 50 µL of 80% TCA (final concentration, 16% TCA). Using the seven absorbance measurements [time zero, (Tz), control growth, (C), and test growth in the presence of drug at the five concentration levels (Ti)], the percentage growth is calculated at each of the drug concentrations levels. Percentage growth inhibition (PGI) is calculated as:

$$ \left[ {\left( {{\text{Ti}} - {\text{Tz}}} \right)/\left( {{\text{C}} - {\text{Tz}}} \right)} \right] \, \times { 1}00 \, \;{\text{for}}\;{\text{ concentrations }}\;{\text{for }}\;{\text{which }}\;{\text{Ti}} > / = {\text{Tz}} $$

$$ \left[ {\left( {{\text{Ti}} - {\text{Tz}}} \right)/{\text{Tz}}} \right] \, \times { 1}00 \, \;{\text{for }}\;{\text{concentrations }}\;{\text{for }}\;{\text{which }}\;{\text{Ti}} < {\text{Tz}}. $$

Three dose response parameters are calculated for each experimental agent. Growth inhibition of 50% (GI50) is calculated from [(Ti − Tz)/(C − Tz)] × 100 = 50, which is the drug concentration resulting in a 50% reduction in the net protein increase (as measured by SRB staining) in control cells during the drug incubation. The drug concentration resulting in total growth inhibition (TGI) is calculated from Ti = Tz. The LC50 (concentration of drug resulting in a 50% reduction in the measured protein at the end of the drug treatment as compared to that at the beginning) indicating a net loss of cells following treatment is calculated from [(Ti − Tz)/Tz] × 100 = − 50. Values are calculated for each of these three parameters if the level of activity is reached; however, if the effect is not reached or is exceeded, the value for that parameter is expressed as greater or less than the maximum or minimum concentration tested.

### Antimicrobial assay

#### Disk-diffusion method

The in vitro antibacterial and antifungal activity of the hybrid quinoline—imidazole/benzimidazole compounds were determined by the Kirby-Bauer disk diffusion method^41^. The method is using Mueller Hinton agar medium for antibacterial assay and Sabouraud nutrient agar medium for antifungal assay. The in vitro antibacterial activity was evaluated against Gram-positive bacteria *Staphylococcus aureus ATCC 25923* and Gram-negative *Escherichia coli ATCC 25922* and the antifungal activity against fungus *Candida albicans* ATCC 10231^23^. Gentamicin was used as positive control (C+) for *S. aureus* and *E. coli* and Nystatin for *C. albicans*. The negative control (C−) consist of sterile filter paper disks (with no antimicrobial compounds) inoculated with DMSO 3%.

For inoculum preparation, reference microbial cultures of bacteria (*Staphylococcus aureus ATCC 25923, Escherichia coli ATCC 25922*) and fungi (*Candida albicans ATCC 10231*) were employed. Approximately 5 colonies from each type of culture were used to inoculate 10 mL of Mueller Hinton (MH) agar (for antibacterial tests) and Sabouraud agar (for antifungal tests). Using a Beckman Coulter DU 730 spectrophotometer (λ = 600 nm), the turbidity of the inoculum was adjusted to a 0.5 McFarland standard (1–2 × 108 CFU/mL for bacteria and 1–5 × 106 CFU/mL for *Candida*), and the inoculum was transferred, in a 1 mL volume, onto the surface of the growth media specific for bacteria (MH) and fungi (Sabouraud). Once the inoculum was absorbed, sterile paper disks of approximately 6 mm in diameter and impregnated with 10 µL of antibacterial compound (dissolved in DMSO 3%) were placed on the surface of the culture media; for all the tested compounds, the concentration used was 25 mg/mL. Following incubation at the optimal temperatures for bacteria and fungi, of 37 °C and 28 °C, respectively, for 24 h (bacteria) and 72 h (fungi), the diameters of the inhibition zones were measured using a ruler. The controls were prepared in the same growth conditions (i.e. C+: sterile filter paper disks impregnated with antibiotics inducing sensitivity in the organisms under investigation, namely Gentamicin 10 μg/mL for *Staphylococcus aureus* and *Escherichia coli* and Nystatin 100 μg/mL for *Candida albicans*, and C-: sterile filter paper disks with no antimicrobial compounds − 10 µL DMSO 3%).

#### Broth microdilution method for determining the minimum inhibitory concentration (MIC)

For the minimum inhibitory concentration (MIC) assay was used the 96-well microtiter plate (microdilution) technique^43,44^. The MIC was evaluated against Gram-positive bacteria *Staphylococcus aureus ATCC 25923* and Gram-negative *Escherichia coli ATCC 25922* and the antifungal activity against fungus *Candida albicans ATCC 10231*^23,35^. For each tested microorganism, a positive control C+ (containing 80 µL of MH growth medium and 10 µL of antimicrobial compound) and a negative one C− (containing 80 µL of MH growth medium and 10 µL of diluted microbial culture) were prepared. The redox dye, resazurin, was used as colorimetric indicator.

The working technique involves the use of a 96-well microtiter plate (microdilution). In each well of the plate, 80 µL of growth medium MH, 10 µL of microbial inoculum (*Staphylococcus aureus ATCC 25923*, *Escherichia coli ATCC 25922*, or *Candida albicans ATCC 10231*) were prepared in the same manner as in the diffusion test (i.e. by diluting the standardized microbial suspension adjusted to a 0.5 McFarland standard), and 100 µL of antimicrobial substance to be tested were transferred by pipetting, in different concentrations. To this purpose, double dilutions of the antimicrobial agent were made in the DMSO 3%, starting with the 25 µg/mL dilution (e.g. 12.5 µg/mL, 6.25 µg/mL, 3.12 µg/mL, 1.56 µg/mL, 0.78 µg/mL and so on). For each tested microorganism, a positive control C+ (containing 80 µL of MH growth medium and 10 µL of antimicrobial compound) and a negative one C− (containing 80 µL of MH growth medium and 10 µL of diluted microbial culture) were prepared. Following the incubation of the microplates at 37 °C for 24 h (for *Staphylococcus aureus ATCC 25923* and *Escherichia coli ATCC 25922*) and at 28 °C for 72 h (for *Candida albicans ATCC 10231*), 10 µL of resazurin were added in each well. The samples were incubated once again at the temperature optimal for each microorganism for one hour. The colour of the indicator turned from purple to pink. Resazurin is a colorimetric indicator for cell viability widely applied for monitoring cell proliferation^46^. The redox dye, resazurin, enters the cytosol in the oxidized form (purple-blue) and is converted to the reduced form, resorufin (pink).

## Supplementary Information

Supplementary Information.

## Data Availability

All data generated or analysed during this study are included in this published article [and its supplementary information files].
