# Supplementary material for: Quinoline–imidazole/benzimidazole derivatives as dual-/multi-targeting hybrids inhibitors with anticancer and antimicrobial activity
Source: Sci Rep. 2022 Oct 10;12:16988. doi: 10.1038/s41598-022-21435-6 (PMC9551061; doi:10.1038/s41598-022-21435-6)
Supplement: Supplementary file 1 — Supplementary Information. [file 41598_2022_21435_MOESM1_ESM.doc]

**Quinoline - imidazole/benzimidazole derivatives as dual- / multi- targeting hybrids inhibitors with anticancer and antimicrobial activity**

Dumitrela Diaconu^2^, Vasilichia Antoci^1^, Violeta Mangalagiu^2^, Dorina Amariucai-Mantu^1^*, Ionel I. Mangalagiu^1,2^*

^1^Alexandru Ioan Cuza University of Iasi, Faculty of Chemistry, 11 Carol I, 700506-Iasi, Romania

^2^Alexandru Ioan Cuza University of Iasi, Institute of Interdisciplinary Research, Department of Exact and Natural Sciences - CERNESIM Centre, 11 Carol I, 700506-Iasi, Romania

^*^Corresponding authors: [dorina.mantu@uaic.ro](mailto:dorina.mantu@uaic.ro) (D. Amariucai-Mantu); [ionelm@uaic.ro](mailto:ionelm@uaic.ro) (I. I. Mangalagiu)

**Supplementary Electronic Information**

**Table of contents**

| **Cell proliferation assay**  **Antibacterial and antifungal assay**  **NMR Spectrum of two QIBS representative compounds** | **S2-S7**  **S8-S10**  **S11-S14** |
| --- | --- |

***Cell proliferation assay***

In **Supplementary Tables 1-4** are presented the results of the NCI 60 anticancer primary single-dose assay for the QIBS salts and QIBC cycloadducts.

**Supplementary Table 1**

Percentage growth inhibition (PGI %, µM)^a^ caused by compounds **7a, 7b, 7c, 7d, 7e, 7f, 7h, 7k, 11a, 11b, 11c, 11d, 11e, 11f, 11g, 11h** and **11k** against an NCI 60 human cancer cell lines in the single-dose assay.

| Cell type | Compound / Growth inhibition percent (PGI%)^a^ | | | | | | | | | | | | | | | | |
| --- | --- | --- | --- | --- | --- | --- | --- | --- | --- | --- | --- | --- | --- | --- | --- | --- | --- |
|  | **7a** | **7b** | **7c** | **7d** | **7e** | **7f** | **7h** | **7k** | **11a** | **11b** | **11c** | **11d** | **11e** | **11f** | **11g** | **11h** | **11k** |
| *Leukemia* |  | | | | | | | | | | | | | | | | |
| CCRF-CEM | 3 | 14 | 6 | 4 | 2 | 0 | 36 | 0 | 11 | 16 | 23 | 5 | 2 | 5 | 13 | **90** | 16 |
| HL-60 (TB) | 5 | 31 | 84 | 17 | 3 | 0 | **86** | 0 | 39 | 73 | 59 | 39 | 0 | 18 | 22 | **100** (37)^b^ | 16 |
| K-562 | 22 | 39 | 10 | 26 | 2 | 8 | 50 | 3 | 62 | 49 | 34 | 28 | 8 | 13 | 40 | **88** | 40 |
| MOLT-4 | 0 | 6 | 1 | 3 | 0 | 0 | 27 | 0 | 8 | 10 | 25 | 8 | 0 | 2 | 13 | **89** | 12 |
| RPMI-8226 | 26 | 40 | 13 | 55 | 0 | 7 | 67 | 5 | **90** | **81** | 47 | 45 | 6 | 21 | 39 | **100** (30)^b^ | 42 |
| SR | 0 | 0 | 0 | 0 | 0 | 0 | 47 | 0 | 39 | 23 | 4 | 0 | 0 | 0 | 10 | **100** (17)^b^ | 15 |
| *Non-small Cell Lung Cancer* |  | | | | | | | | | | | | | | | | |
| A549/ATCC | 6 | 11 | 3 | 15 | 0 | 0 | 28 | 0 | **86** | 0 | 6 | 0 | 0 | 0 | 0 | 41 | 0 |
| EKVX | 14 | 22 | 13 | 20 | 21 | 12 | 24 | 7 | 27 | 6 | 11 | 5 | 9 | 10 | 10 | 53 | 25 |
| HOP-62 | 1 | 1 | 0 | 8 | 0 | 0 | 22 | 4 | 5 | 10 | 0 | 4 | 1 | 1 | 12 | 57 | 10 |
| HOP-92 | 30 | 35 | 19 | 49 | 31 | 10 | 27 | 0 | 73 | 61 | 16 | 33 | 20 | 35 | 14 | **100** (4)^b^ | 61 |
| NCI-H226 | 13 | 5 | 13 | 9 | 10 | 5 | 34 | 1 | 20 | 8 | 12 | 4 | 7 | 4 | 8 | 50 | 10 |
| NCI-H23 | 16 | 25 | 10 | 16 | 2 | 2 | 5 | 3 | 50 | 17 | 9 | 5 | 3 | 1 | 14 | 77 | 16 |
| NCI-H322M | 8 | 7 | 2 | 7 | 5 | 1 | 13 | 0 | 5 | 3 | 2 | 5 | 3 | 6 | 1 | 23 | 13 |
| NCI-460 | 1 | 2 | 11 | 8 | 0 | 1 | 58 | 0 | 23 | 3 | 25 | 2 | 0 | 0 | 0 | 78 | 0 |
| NCI-H522 | 15 | 22 | 34 | 19 | 7 | 10 | 26 | 8 | 53 | 56 | 24 | 27 | 12 | 18 | 36 | **98** | 23 |
| *Colon Cancer* |  | | | | | | | | | | | | | | | | |
| COLO 205 | 4 | 30 | 2 | 16 | 0 | 0 | 36 | 0 | 75 | 40 | 24 | 22 | 0 | 3 | 21 | **100** (61)^b^ | 10 |
| HCC-2998 | 0 | 16 | 4 | 15 | 0 | 12 | 49 | 0 | 50 | 30 | 15 | 5 | 0 | 3 | 7 | **94** | 6 |
| HCT-116 | 8 | 17 | 1 | 15 | 0 | 5 | 38 | 0 | 63 | 17 | 8 | 16 | 0 | 0 | 6 | **89** | 16 |
| HT29 | 7 | 20 | 0 | 19 | 0 | 3 | 41 | 0 | 60 | 37 | 16 | 59 | 0 | 5 | 19 | **93** | 11 |
| KM12 | 1 | 8 | 9 | 3 | 0 | 1 | 69 | 1 | 22 | 13 | 23 | 2 | 0 | 1 | 6 | **100 (31)^b^** | 1 |
| SW-620 | 5 | 3 | 6 | 6 | 3 | 3 | 16 | 0 | 46 | 7 | 8 | 9 | 2 | 6 | 6 | 64 | 0 |
| *CNS Cancer* |  | | | | | | | | | | | | | | | | |
| SF-268 | 12 | 22 | 13 | 18 | 7 | 10 | 35 | 7 | 35 | 31 | 30 | 32 | 17 | 29 | 28 | **80** | 25 |
| SF-295 | 0 | 1 | 3 | 0 | 0 | 0 | 0 | 1 | 0 | 1 | 5 | 0 | 2 | 5 | 0 | 22 | 0 |
| SF-539 | 9 | 19 | 1 | 12 | 14 | 5 | 21 | 2 | 13 | 19 | 10 | 4 | 0 | 9 | 8 | 77 | 16 |
| SNB-19 | 16 | 23 | 12 | 20 | 6 | 6 | 43 | 5 | 63 | 32 | 17 | 23 | 10 | 17 | 21 | **93** | 14 |
| SNB-75 | 42 | 55 | 43 | 55 | **88** | 18 | 37 | 10 | 71 | 65 | 52 | **81** | 49 | 63 | 48 | **100** (6)^b^ | 61 |
| U251 | 0 | 14 | 9 | 8 | 0 | 2 | 42 | 4 | 37 | 14 | 15 | 6 | 0 | 11 | 9 | **80** | 7 |
| *Melanoma* |  | | | | | | | | | | | | | | | | |
| LOX IMVI | 15 | 13 | 5 | 13 | 9 | 3 | 31 | 4 | 14 | 10 | 10 | 9 | 5 | 10 | 9 | **100** (45)^b^ | 16 |
| MALME-3M | 18 | 20 | 2 | 17 | 14 | 3 | 16 | 0 | 19 | 14 | 8 | 13 | 4 | 8 | 13 | **92** | 27 |
| M14 | 6 | 11 | 0 | 7 | 5 | 0 | 21 | 0 | 22 | 16 | 3 | 0 | 2 | 1 | 1 | 72 | 15 |
| MDA-MB-435 | 0 | 6 | 0 | 2 | 1 | 0 | 18 | 0 | 40 | 18 | 13 | 7 | 0 | 4 | 5 | **93** | 1 |
| SK-MEL-2 | 7 | 13 | 0 | 9 | 1 | 0 | 26 | 0 | 60 | 26 | 23 | 4 | 0 | 4 | 12 | **81** | 16 |
| SK-MEL-28 | 0 | 7 | 8 | 4 | 0 | 0 | 31 | 0 | 22 | 31 | 26 | 20 | 0 | 10 | 9 | 79 | 3 |
| SK-MEL-5 | 12 | 15 | 8 | 12 | 4 | 1 | 66 | 0 | 5 | 3 | 44 | 2 | 1 | 6 | 4 | **100** (35)^b^ | 9 |
| UACC-257 | 17 | 24 | 15 | 17 | 0 | 0 | 41 | 0 | 52 | 63 | 33 | 17 | 0 | 7 | 31 | **90** | 8 |
| UACC-62 | 13 | 23 | 41 | 20 | 4 | 1 | 32 | 1 | 31 | 43 | 21 | 23 | 11 | 15 | 34 | **100** (32)^b^ | 26 |
| *Ovarian Cancer* |  | | | | | | | | | | | | | | | | |
| IGROV1 | 10 | 23 | 37 | 14 | 9 | 8 | **73** | 5 | 29 | 42 | 50 | 51 | 11 | 28 | 53 | **100** (38)^b^ | 32 |
| OVCAR-3 | 7 | 32 | 20 | 37 | 0 | 0 | 50 | 0 | 62 | 58 | 39 | 38 | 0 | 19 | 33 | **100** (1)^b^ | 26 |
| OVCAR-4 | 23 | 57 | 18 | 56 | 18 | 0 | 68 | 0 | **80** | 76 | 41 | 26 | 3 | 15 | 26 | **90** | 35 |
| OVCAR-5 | 3 | 2 | 0 | 11 | 0 | 0 | 10 | 0 | 7 | 10 | 9 | 8 | 2 | 6 | 1 | 38 | 12 |
| OVCAR-8 | 2 | 8 | 2 | 4 | 0 | 0 | 24 | 0 | 7 | 3 | 6 | 0 | 0 | 0 | 3 | **80** | 5 |
| NCI/ADR-RES | 4 | 0 | 1 | 4 | 0 | 1 | 3 | 0 | 0 | 0 | 3 | 0 | 0 | 3 | 0 | 4 | 5 |
| SK-OV-3 | 0 | 10 | 21 | 1 | 0 | 0 | 20 | 0 | 0 | 0 | 10 | 0 | 0 | 0 | 12 | 50 | 11 |
| *Renal Cancer* |  | | | | | | | | | | | | | | | | |
| 786-0 | 27 | 31 | 0 | 36 | 18 | 0 | 5 | 0 | 6 | 6 | 0 | 0 | 0 | 2 | 0 | 56 | 0 |
| ACHN | 0 | 0 | 0 | 0 | 0 | 0 | 12 | 0 | 7 | 1 | 13 | 6 | 1 | 0 | 0 | 10 | 2 |
| CAKI-1 | 11 | 7 | 3 | 8 | 14 | 11 | 0 | 4 | 16 | 4 | 0 | 3 | 8 | 4 | 9 | 56 | 13 |
| RXF 393 | 19 | 8 | 12 | 10 | 0 | 0 | 0 | 0 | 3 | 6 | 3 | 0 | 6 | 4 | 0 | 17 | 2 |
| SN12C | 12 | 20 | 4 | 11 | 4 | 0 | 15 | 1 | 71 | 17 | 0 | 14 | 9 | 16 | 12 | **86** | 17 |
| TK-10 | 0 | 0 | 0 | 0 | 0 | 0 | 0 | 0 | 0 | 4 | 14 | 3 | 0 | 1 | 0 | 12 | 2 |
| UO-31 | 21 | 27 | 20 | 18 | 19 | 19 | 6 | 16 | 35 | 21 | 9 | 27 | 29 | 17 | 27 | 33 | 36 |
| *Prostate Cancer* |  | | | | | | | | | | | | | | | | |
| PC-3 | 12 | 24 | 19 | 17 | 5 | 0 | 46 | 1 | 62 | 33 | 28 | 17 | 10 | 12 | 29 | **88** | 23 |
| DU-145 | 0 | 15 | 0 | 8 | 1 | 0 | 24 | 0 | 15 | 2 | 3 | 0 | 10 | 0 | 0 | 64 | 9 |
| *Breast Cancer* |  | | | | | | | | | | | | | | | | |
| MCF7 | 14 | 24 | 34 | 20 | 8 | 11 | 50 | 11 | 70 | 25 | 29 | 13 | 12 | 8 | 14 | **88** | 16 |
| MDA-MB-231/ATCC | 19 | 19 | 32 | 13 | 17 | 6 | 27 | 4 | 50 | 11 | 21 | 13 | 11 | 4 | 22 | **100** (7)^b^ | 30 |
| HS 578T | 8 | 7 | 2 | 7 | 8 | 4 | 25 | 0 | 26 | 12 | 15 | 15 | 4 | 15 | 8 | 67 | 0 |
| BT-549 | 14 | 29 | 5 | 22 | 6 | 0 | 43 | 0 | 65 | 56 | 23 | 25 | 3 | 26 | 3 | **100** (9)^b^ | 20 |
| T-47D | 11 | 26 | 17 | 21 | 0 | 13 | 60 | 8 | 67 | 66 | 33 | 25 | 1 | 10 | 27 | **90** | 31 |
| MDA-MB-468 | 53 | 56 | 29 | 50 | 14 | 3 | **92** | 0 | **91** | **86** | 70 | 51 | 23 | 30 | 68 | **100** (6)^b^ | 42 |

^a^ The number reported for the one-dose assay, percentage growth inhibition (PGI), is growth relative to the no-drug control, and relative to the time zero number of cells; ^b^ Cytotoxic effect; lethality percent is represented in brackets; the most active compounds are highlighted in bold and red

**Supplementary Table 2**

Percentage growth inhibition (PGI %, µM)^a^ caused by compounds **8a-k** against an NCI 60 human cancer cell lines in the single-dose assay.

| Cell type |  |  | Compound / Growth inhibition percent (PGI%)^a^ | | | | | | | | |
| --- | --- | --- | --- | --- | --- | --- | --- | --- | --- | --- | --- |
|  | **8a** | **8b** | **8c** | **8d** | **8e** | **8f** | **8g** | **8h** | **8i** | **8j** | **8k** |
| *Leukemia* |  | |  | | | | | | | | |
| CCRF-CEM | 50 | 14 | 3 | 3 | 0 | 2 | 4 | 22 | 5 | 0 | 0 |
| HL-60 (TB) | 0 | 10 | 12 | - | 4 | 30 | 20 | **87** | 43 | 30 | 32 |
| K-562 | 13 | 23 | 11 | - | 0 | 7 | 16 | 55 | 14 | 9 | 5 |
| MOLT-4 | 0 | 4 | 2 | - | 0 | 0 | 6 | 16 | 5 | 0 | 0 |
| RPMI-8226 | 13 | 36 | 24 | 17 | 0 | 0 | 13 | **66** | 6 | 3 | 2 |
| SR | 0 | 0 | 0 | - | 3 | 0 | 0 | 21 | 0 | 0 | 0 |
| *Non-Small Cell Lung Cancer* |  | |  | | | | | | | | |
| A549/ATCC | 5 | 1 | 2 | 4 | 0 | 3 | 2 | 6 | 0 | 1 | 5 |
| EKVX | 8 | 15 | 8 | 10 | 4 | 3 | 10 | 19 | 8 | 2 | 6 |
| HOP-62 | 0 | 0 | 0 | 0 | 0 | 0 | 0 | 0 | 0 | 0 | 0 |
| NCI-H226 | 11 | 19 | 13 | 5 | 3 | 4 | 8 | 17 | 7 | 2 | 4 |
| NCI-H23 | 4 | 14 | 10 | 11 | 6 | 7 | 12 | 19 | 10 | 7 | 4 |
| NCI-H322M | 5 | 6 | 3 | 5 | 0 | 2 | 4 | 8 | 5 | 2 | 3 |
| NCI-460 | 0 | 0 | 1 | 0 | 7 | 0 | 0 | 9 | 0 | 0 | 0 |
| NCI-H522 | 5 | 14 | 10 | 14 | 4 | 5 | 13 | 53 | 8 | 4 | 6 |
| *Colon Cancer* |  | |  | | | | | | | | |
| COLO 205 | 0 | 10 | 4 | 9 | 0 | 0 | 7 | 28 | 6 | 0 | 0 |
| HCC-2998 | 5 | 1 | 1 | 5 | 0 | 0 | 1 | 32 | 4 | 0 | 0 |
| HCT-116 | 5 | 10 | 8 | 5 | 0 | 0 | 6 | 16 | 6 | 0 | 0 |
| HCT-15 | 6 | 0 | 0 | 7 | 5 | 7 | 3 | 4 | 4 | 6 | 3 |
| HT29 | 0 | 18 | 10 | 13 | 0 | 0 | 21 | 30 | 4 | 0 | 0 |
| KM12 | 4 | 6 | 5 | 7 | 0 | 7 | 7 | **64** | 9 | 8 | 2 |
| SW-620 | 10 | 9 | 8 | 5 | 2 | 2 | 8 | 15 | 6 | 6 | 1 |
| *CNS Cancer* |  | |  | | | | | | | | |
| SF-268 | 2 | 11 | 13 | 10 | 2 | 5 | 13 | 18 | 6 | 7 | 6 |
| SF-295 | 1 | 0 | 1 | 6 | 5 | 7 | 9 | 0 | 6 | 4 | 4 |
| SF-539 | 4 | 11 | 5 | 1 | 0 | 0 | 0 | 20 | 0 | 0 | 0 |
| SNB-19 | 11 | 8 | 9 | 11 | 0 | 3 | 15 | 40 | 12 | 8 | 5 |
| SNB-75 | 24 | 1 | 6 | 16 | 14 | 18 | 27 | 55 | 23 | 16 | 14 |
| U251 | 10 | 0 | 4 | 7 | 2 | 1 | 9 | 40 | 7 | 6 | 0 |
| *Melanoma* |  | |  | | | | | | | | |
| LOX IMVI | 6 | 6 | 8 | 3 | 1 | 0 | 2 | 18 | 3 | 4 | 3 |
| M14 | 5 | 3 | 6 | 0 | 2 | 2 | 4 | 18 | 1 | 3 | 0 |
| MDA-MB-435 | 0 | 0 | 0 | 0 | 0 | 0 | 0 | 13 | 0 | 0 | 0 |
| SK-MEL-2 | 0 | 0 | 0 | 0 | 0 | 0 | 0 | 10 | 0 | 0 | 0 |
| SK-MEL-28 | 0 | 0 | 0 | 0 | 2 | 0 | 0 | 33 | 2 | 0 | 3 |
| SK-MEL-5 | 2 | 2 | 2 | 6 | 3 | 4 | 9 | 19 | 1 | 6 | 8 |
| UACC-257 | 10 | 12 | 4 | 16 | 8 | 7 | 3 | 60 | 9 | 9 | 7 |
| UACC-62 | 11 | 12 | 7 | 12 | 0 | 7 | 11 | 42 | 9 | 8 | 1 |
| *Ovarian Cancer* |  | | | |  | | | | | | |
| IGROV1 | 1 | 20 | 18 | 13 | 1 | 2 | 17 | **64** | 9 | 0 | 0 |
| OVCAR-3 | 7 | 16 | 7 | 20 | 0 | 0 | 7 | 49 | 7 | 1 | 8 |
| OVCAR-4 | 28 | 50 | 16 | 23 | 0 | 6 | 19 | **86** | 6 | 10 | 7 |
| OVCAR-5 | 0 | 6 | 8 | 5 | 0 | 0 | 4 | 9 | 6 | 1 | 1 |
| OVCAR-8 | 4 | 2 | 3 | 2 | 1 | 0 | 3 | 18 | 2 | 3 | 0 |
| NCI/ADR-RES | 0 | 5 | 4 | 3 | 1 | 3 | 1 | 1 | 1 | 0 | 2 |
| SK-OV-3 | 6 | 13 | 16 | 5 | 0 | 7 | 8 | 26 | 8 | 4 | 0 |
| *Renal Cancer* |  |  |  | | | | | | | | |
| 786-0 | 3 | 1 | 2 | 0 | 0 | 0 | 0 | 3 | 2 | 0 | 0 |
| ACHN | 8 | 2 | 5 | 12 | 12 | 16 | 0 | 2 | 23 | 9 | 15 |
| SN12C | 9 | 8 | 4 | 0 | 0 | 0 | 0 | 10 | 0 | 0 | 0 |
| TK-10 | 0 | 0 | 0 | 2 | 0 | 0 | 1 | 13 | 3 | 0 | 3 |
| UO-31 | 11 | 10 | 12 | 9 | 11 | 8 | 10 | 2 | 10 | 9 | 9 |
| *Prostate Cancer* |  | |  | | | | | | | | |
| PC-3 | 12 | 18 | 17 | 10 | 0 | 9 | 14 | 45 | 11 | 6 | 6 |
| DU-145 | 0 | 2 | 3 | 1 | 0 | 0 | 0 | 10 | 0 | 0 | 0 |
| *Breast Cancer* |  | |  | | | | | | | | |
| MCF7 | 15 | 14 | 16 | 2 | 17 | 16 | 21 | 42 | 17 | 16 | 14 |
| MDA-MB-231/ATCC | 22 | 2 | 10 | 10 | 3 | 6 | 10 | 35 | 9 | 9 | 0 |
| HS 578T | 1 | 5 | 7 | 7 | 2 | 7 | 6 | 29 | 3 | 7 | 2 |
| BT-549 | 17 | 7 | 0 | 6 | 0 | 0 | 1 | 43 | 0 | 0 | 1 |
| T-47D | 10 | 12 | 5 | 3 | 3 | 2 | 5 | 58 | 5 | 5 | 1 |
| MDA-MB-468 | 57 | **70** | 38 | **72** | 9 | 20 | 40 | **100**(26)^b^ | 40 | 20 | 16 |

^a^ The number reported for the one-dose assay, percentage growth inhibition (PGI), is growth relative to the no-drug control, and relative to the time zero number of cells; ^b^ Cytotoxic effect; lethality percent is represented in brackets; the most active compounds are highlighted in bold and red

**Supplementary Table 3**

Percentage growth inhibition (PGI %, µM)^a^ caused by compounds **12a-k** against an NCI 60 human cancer cell lines in the single-dose assay.

| Cell type | Compound / Growth inhibition percent (PGI%)^a^ | | | | | | | | | | |
| --- | --- | --- | --- | --- | --- | --- | --- | --- | --- | --- | --- |
|  | **12a** | **12b** | **12c** | **12d** | **12e** | **12f** | **12g** | **12h** | **12i** | **12j** | **12k** |
| *Leukemia* |  | | | | | | | | | | |
| CCRF-CEM | 12 | 6 | 0 | 4 | 0 | 32 | 4 | 64 | 16 | 0 | 0 |
| HL-60 (TB) | 13 | 32 | 0 | 10 | 0 | 64 | 0 | **100**(42)^b^ | 27 | 31 | 0 |
| K-562 | 50 | 30 | 0 | 24 | 0 | 42 | 16 | **78** | 22 | 4 | 4 |
| MOLT-4 | 2 | 2 | 0 | 4 | 0 | 35 | 2 | 53 | 16 | 8 | 0 |
| RPMI-8226 | 57 | 45 | 0 | 22 | 0 | **75** | 13 | **97** | 13 | 1 | 7 |
| SR | 0 | 0 | 0 | 0 | 0 | 21 | 0 | **91** | 0 | 0 | 0 |
| *Non-Small Cell Lung Cancer* |  | | | | | | | | | | |
| EKVX | 12 | 4 | 0 | 0 | 1 | 11 | 5 | 39 | 10 | 4 | 0 |
| HOP-62 | 0 | 0 | 0 | 0 | 0 | 15 | 15 | 6 | 7 | 5 | 0 |
| NCI-H226 | 9 | 0 | 0 | 3 | 0 | 22 | 17 | 43 | 11 | 7 | 5 |
| NCI-H23 | 13 | 0 | 0 | 6 | 4 | 17 | 7 | 40 | 11 | 7 | 7 |
| NCI-H322M | 4 | 13 | 0 | 0 | 4 | 7 | 10 | 18 | 11 | 14 | 2 |
| NCI-460 | 6 | 0 | 0 | 0 | 0 | 12 | 10 | 45 | 1 | 0 | 2 |
| NCI-H522 | 11 | 10 | 0 | 4 | 0 | 33 | 13 | 53 | 18 | 9 | 12 |
| *Colon Cancer* |  | | | | | | | | | | |
| COLO 205 | 24 | 19 | 0 | 7 | 1 | 45 | 13 | 56 | 15 | 13 | 9 |
| HCC-2998 | 24 | 1 | 0 | 0 | 3 | 14 | 6 | **62** | 11 | 5 | 0 |
| HCT-116 | 18 | 18 | 0 | 0 | 11 | 47 | 16 | 44 | 27 | 8 | 6 |
| HCT-15 | 4 | 0 | 0 | 2 | 0 | 0 | 0 | 7 | 5 | 2 | 0 |
| HT29 | 14 | 17 | 0 | 0 | 0 | 42 | 7 | 49 | 15 | 5 | 8 |
| KM12 | 8 | 4 | 0 | 2 | 0 | 34 | 3 | **86** | 4 | 6 | 2 |
| SW-620 | 20 | 0 | 0 | 4 | 0 | 8 | 11 | 35 | 10 | 3 | 6 |
| *CNS Cancer* |  | | | | | | | | | | |
| SF-268 | 17 | 0 | 0 | 7 | 0 | 32 | 8 | 45 | 13 | 4 | 9 |
| SF-539 | 0 | 0 | 0 | 0 | 0 | 14 | 2 | 25 | 3 | 6 | 2 |
| SNB-19 | 19 | 3 | 0 | 2 | 7 | 39 | 7 | 59 | 7 | 2 | 7 |
| SNB-75 | 18 | 0 | 0 | 0 | 51 | 35 | 11 | 59 | 42 | 21 | 6 |
| U251 | 15 | 6 | 0 | 2 | 0 | 31 | 8 | 58 | 12 | 7 | 6 |
| *Melanoma* |  | | | | | | | | | | |
| LOX IMVI | 4 | 0 | 0 | 4 | 2 | 16 | 8 | 43 | 11 | 6 | 12 |
| M14 | 7 | 0 | 0 | 0 | 1 | 21 | 3 | 51 | 7 | 4 | 4 |
| MDA-MB-435 | 4 | 0 | 0 | 0 | 1 | 22 | 0 | 68 | 3 | 0 | 0 |
| SK-MEL-2 | 13 | 3 | 0 | 0 | 1 | 19 | 3 | 35 | 0 | 0 | 4 |
| SK-MEL-28 | 16 | 14 | 0 | 9 | 3 | 40 | 6 | 62 | 10 | 2 | 6 |
| SK-MEL-5 | 7 | 27 | 3 | 12 | 0 | 67 | 8 | **80** | 4 | 6 | 11 |
| UACC-257 | 20 | 37 | 0 | 1 | 0 | 53 | 7 | **77** | 15 | 9 | 8 |
| UACC-62 | 6 | 9 | 0 | 3 | 1 | 45 | 6 | **85** | 17 | 6 | 3 |
| *Ovarian Cancer* |  | | | | | | | | | | |
| IGROV1 | 0 | 18 | 0 | 23 | 6 | 60 | 18 | **90** | 30 | 5 | 10 |
| OVCAR-3 | 45 | 24 | 0 | 10 | 0 | 45 | 10 | **72** | 12 | 2 | 8 |
| OVCAR-4 | 58 | 47 | 0 | 20 | 2 | 48 | 25 | **76** | 14 | 17 | 16 |
| OVCAR-5 | 4 | 9 | 0 | 7 | 2 | 21 | 12 | 19 | 14 | 7 | 7 |
| OVCAR-8 | 1 | 3 | 0 | 0 | 0 | 14 | 5 | 48 | 4 | 0 | 0 |
| SK-OV-3 | 0 | 2 | 0 | 1 | 0 | 12 | 9 | 26 | 8 | 0 | 0 |
| *Renal Cancer* |  | | | | | | | | | | |
| 786-0 | 0 | 0 | 0 | 0 | 0 | 0 | 0 | 29 | 1 | 0 | 0 |
| ACHN | 14 | 0 | 1 | 0 | 0 | 0 | 15 | 12 | 4 | 26 | 7 |
| RXF 393 | 0 | 0 | 0 | 0 | 0 | 1 | 5 | 23 | 2 | 0 | 0 |
| SN12C | 0 | 0 | 0 | 0 | 0 | 8 | 0 | 31 | 2 | 0 | 1 |
| TK-10 | 5 | 0 | 0 | 0 | 0 | 7 | 0 | 47 | 6 | 1 | 0 |
| UO-31 | 7 | 4 | 4 | 3 | 0 | 12 | 13 | 18 | 18 | 12 | 6 |
| *Prostate Cancer* |  | | | | | | | | | | |
| PC-3 | 19 | 13 | 0 | 3 | 4 | 37 | 19 | **70** | 35 | 7 | 6 |
| DU-145 | 5 | 0 | 0 | 0 | 0 | 4 | 0 | 22 | 0 | 0 | 0 |
| *Breast Cancer* |  | | | | | | | | | | |
| MCF7 | 60 | 15 | 0 | 11 | 3 | 32 | 13 | **81** | 35 | 12 | 2 |
| MDA-MB-231/ATCC | 24 | 2 | 0 | 11 | 7 | 43 | 20 | 51 | 19 | 0 | 11 |
| HS 578T | 0 | 0 | 0 | 8 | 0 | 26 | 14 | 48 | 14 | 10 | 18 |
| BT-549 | 14 | 0 | 0 | 0 | 10 | 31 | 18 | 47 | 14 | 12 | 15 |
| T-47D | 12 | 31 | 0 | 9 | 10 | 47 | 16 | **80** | 14 | 18 | 6 |
| MDA-MB-468 | **74** | **70** | 0 | 40 | 12 | **100**(17)^b^ | 31 | **100**(32)^b^ | 48 | 30 | 33 |

^a^ The number reported for the one-dose assay, percentage growth inhibition (PGI), is growth relative to the no-drug control, and relative to the time zero number of cells; ^b^ Cytotoxic effect; lethality percent is represented in brackets; the most active compounds are highlighted in bold and red

**Supplementary Table 4**

Percentage growth inhibition (PGI %, µM)^a^ caused by compounds **13a**, **13c**, **13d**, **13e**, **13f**, **13g** and **13h** against an NCI 60 human cancer cell lines in the single-dose assay.

| Cell type | Compound / Growth inhibition percent (PGI%)^a^ | | | | | | |
| --- | --- | --- | --- | --- | --- | --- | --- |
|  | **13a** | **13c** | **13d** | **13e** | **13f** | **13g** | **13h** |
| *Leukemia* |  | | | | | | |
| CCRF-CEM | 0 | 0 | 5 | 1 | 0 | 37 | 0 |
| HL-60 (TB) | 3 | 5 | 1 | 0 | 1 | 24 | 0 |
| K-562 | 5 | 11 | 9 | 7 | 1 | 21 | 4 |
| MOLT-4 | 0 | 7 | 7 | 4 | 4 | 38 | 0 |
| RPMI-8226 | 0 | 0 | 0 | 6 | 4 | 0 | 0 |
| SR | 0 | 0 | 0 | 0 | 0 | 0 | 0 |
| *Non-Small Cell Lung Cancer* |  | | | | | | |
| A549/ATCC | 0 | 0 | 9 | 1 | 1 | 4 | 0 |
| EKVX | 0 | 0 | 3 | 0 | 0 | 0 | 7 |
| HOP-62 | 3 | 1 | 4 | 0 | 3 | 17 | 0 |
| NCI-H226 | 0 | 2 | 11 | 16 | 12 | 19 | 17 |
| NCI-H23 | 0 | 11 | 13 | 3 | 0 | 3 | 6 |
| NCI-H322M | 3 | 3 | 6 | 0 | 0 | 0 | 3 |
| NCI-460 | 4 | 5 | 1 | 0 | 0 | 0 | 0 |
| NCI-H522 | 11 | 10 | 20 | 4 | 5 | 3 | 2 |
| *Colon Cancer* |  | | | | | | |
| COLO 205 | 0 | 0 | 0 | 0 | 0 | 0 | 0 |
| HCC-2998 | 0 | 0 | 0 | 0 | 0 | 0 | 0 |
| HCT-116 | 12 | 0 | **51** | 0 | 0 | 3 | 0 |
| HCT-15 | 0 | 0 | 3 | 1 | 3 | 0 | 1 |
| HT29 | 0 | 0 | 0 | 0 | 0 | 0 | 0 |
| KM12 | 0 | 3 | 0 | 3 | 0 | 20 | 2 |
| SW-620 | 0 | 3 | 5 | 2 | 0 | 2 | 2 |
| *CNS Cancer* |  | | | | | | |
| SF-268 | 0 | 3 | 3 | 5 | 0 | 0 | 3 |
| SF-295 | 0 | 0 | 5 | 0 | 0 | 4 | 3 |
| SF-539 | 0 | 3 | 0 | 0 | 0 | 1 | 0 |
| SNB-19 | 6 | 5 | 9 | 4 | 4 | 14 | 0 |
| SNB-75 | 4 | 1 | 0 | 0 | 11 | 1 | 19 |
| U251 | 1 | 0 | 3 | 1 | 0 | 0 | 1 |
| *Melanoma* |  | | | | | | |
| LOX IMVI | 4 | 6 | 2 | 7 | 1 | 16 | 3 |
| M14 | 4 | 7 | 3 | 0 | 0 | 0 | 1 |
| SK-MEL-5 | 0 | 6 | 4 | 10 | 3 | 6 | 5 |
| UACC-257 | 0 | 0 | 0 | 0 | 0 | 0 | 0 |
| UACC-62 | 9 | 14 | 16 | 7 | 9 | 3 | 11 |
| *Ovarian Cancer* |  | | | | | | |
| IGROV1 | 0 | 3 | 3 | 0 | 1 | 5 | 0 |
| OVCAR-3 | 0 | 0 | 0 | 1 | 0 | 4 | 4 |
| OVCAR-4 | 0 | 1 | 1 | 5 | 7 | 12 | 0 |
| OVCAR-5 | 0 | 0 | 0 | 4 | 6 | 0 | 1 |
| OVCAR-8 | 0 | 0 | 8 | 3 | 2 | 13 | 2 |
| NCI/ADR-RES | 0 | 0 | 5 | 7 | 7 | 13 | 5 |
| SK-OV-3 | 0 | 1 | 0 | 0 | 6 | 0 | 4 |
| *Renal Cancer* |  | | | | | | |
| 786-0 | 0 | 0 | 0 | 0 | 0 | 0 | 0 |
| ACHN | 0 | 0 | 0 | 5 | 0 | 0 | 4 |
| CAKI-1 | 2 | 5 | 3 | 10 | 8 | 6 | 1 |
| RXF 393 | 0 | 1 | 0 | 9 | 9 | 0 | 9 |
| SN12C | 0 | 0 | 3 | 0 | 0 | 1 | 0 |
| TK-10 | 5 | 3 | 8 | 0 | 0 | 0 | 2 |
| UO-31 | 16 | 24 | 25 | 17 | 17 | 14 | 26 |
| *Prostate Cancer* |  | | | | | | |
| PC-3 | 10 | 13 | 18 | 10 | 7 | 24 | 15 |
| DU-145 | 0 | 0 | 0 | 0 | 0 | 8 | 0 |
| *Breast Cancer* |  | | | | | | |
| MCF7 | 9 | 8 | 7 | 1 | 0 | 13 | 13 |
| MDA-MB-231/ATCC | 6 | 10 | 12 | 10 | 8 | 7 | 10 |
| HS 578T | 0 | 0 | 0 | 0 | 0 | 3 | 0 |
| BT-549 | 0 | 0 | 0 | 0 | 0 | 8 | 0 |
| T-47D | 1 | 2 | 17 | 9 | 3 | 12 | 3 |
| MDA-MB-468 | 0 | 0 | 2 | 0 | 5 | 2 | 7 |

^a^ The number reported for the one-dose assay, percentage growth inhibition (PGI), is growth relative to the no-drug control, and relative to the time zero number of cells; the most active compounds are highlighted in bold and red

.***Antibacterial and antifungal assay***

**Supplementary Table 5**

**The antibacterial and antifungal activity assay for the hybrid quinoline - imidazole/benzimidazole compounds 7a-k, determined by**[**disk diffusion**](https://www.sciencedirect.com/topics/biochemistry-genetics-and-molecular-biology/disk-diffusion)**assay.**

| **Strain** | **Compound / Diameter of inhibition zone (mm)** | | | | | | | | | | | | | |
| --- | --- | --- | --- | --- | --- | --- | --- | --- | --- | --- | --- | --- | --- | --- |
|  | **7a** | **7b** | **7c** | **7d** | **7e** | **7f** | **7g** | **7h** | **7i** | **7j** | **7k** | C+_G_ | C+_N_ | C- |
| *S. aureus* | 8±1.3 | 10±1.5 | 15±1.3 | 14±0.5 | 5.5±1 | 5±0.5 | 13.5±1.2 | 6±0.8 | 16±1 | 12±1 | 18±1 | 14±1.4 | X | 0 |
| *E. coli* | 14.5±1 | 15.5±2 | 12±1.7 | 18.5±1 | 10±1 | 8±1 | 16±2.3 | 11±1.2 | 17±1 | 18.5±1 | 15.5±1 | 12±1.1 | X | 0 |
| *C.albicans* | 0 | 7 | 20.5±1 | 10±1 | 6±1 | 11±1 | 0 | 10±1 | 15±1 | 0±1 | 19.5±1 | X | 27±1.8 | 0 |

*S. aureus: Staphylococcus. aureus ATCC 25923; E. coli: Escherichia coli ATCC 25922; C. albicans: Candida albicans ATCC 10231; X±SD, mean of three measurements ± standard deviation*

C+_G_= Gentamicin, C+_N_= Nistatin, C-= DMSO 3%

**Supplementary Table 6**

The antibacterial and antifungal activity assay for the hybrid quinoline - imidazole/benzimidazole compounds **8a-k**, determined by [disk diffusion](https://www.sciencedirect.com/topics/biochemistry-genetics-and-molecular-biology/disk-diffusion) assay.

| **Strain** | **Compound / Diameter of inhibition zone (mm)** | | | | | | | | | | | | | |
| --- | --- | --- | --- | --- | --- | --- | --- | --- | --- | --- | --- | --- | --- | --- |
|  | **8a** | **8b** | **8c** | **8d** | **8e** | **8f** | **8g** | **8h** | **8i** | **8j** | **8k** | C+_G_ | C+_N_ | C- |
| *S. aureus* | 6±0.7 | 6±1 | 12±1.6 | 11±2 | 0 | 6±1.1 | 10±1.3 | 8±1 | 20±1.3 | 14±1.1 | 9±1 | 14±1.4 | X | 0 |
| *E. coli* | 11±1.1 | 12±1.5 | 15±1 | 17±0.7 | 0 | 11±1.8 | 16±1.5 | 18±2 | 12±0.7 | 6±0.5 | 0 | 12±1.1 | X | 0 |
| *C. albicans* | 0 | 0 | 0 | 0 | 0 | 0 | 0 | 0 | 0 | 0 | 0 | X | 27±1.8 | 0 |

*S. aureus: Staphylococcus. aureus ATCC 25923; E. coli: Escherichia coli ATCC 25922; C. albicans: Candida albicans ATCC 10231, X±SD, mean of three mesearurements ± standard deviation*

C+_G_= Gentamicin, C+_N_= Nistatin, C-= DMSO 3%

**Supplementary Table 7**

The antibacterial and antifungal activity assay for the hybrid quinoline - imidazole/benzimidazole compounds **11a-k**, determined by [disk diffusion](https://www.sciencedirect.com/topics/biochemistry-genetics-and-molecular-biology/disk-diffusion) assay.

| **Strain** | **Compound / Diameter of inhibition zone (mm)** | | | | | | | | | | | | | |
| --- | --- | --- | --- | --- | --- | --- | --- | --- | --- | --- | --- | --- | --- | --- |
|  | **11a** | **11b** | **11c** | **11d** | **11e** | **11f** | **11g** | **11h** | **11i** | **11j** | **11k** | C+_G_ | C+_N_ | C- |
| *S. aureus* | 5±1.2 | 11±1.5 | 5±1.6 | 14.5±1 | 7±1 | 16.5±1.6 | 13±1.2 | 7±1.1 | 6±0.8 | 8±1.2 | 9±1.1 | 14±1.4 | X | 0 |
| *E. coli* | 12±0.8 | 16±1.8 | 6±0.5 | 17±1.4 | 11.5±1.8 | 18±1 | 16±2 | 11±1.5 | 7±1 | 14±1.1 | 6±1 | 12±1.1 | X | 0 |
| *C.albicans* | 13±1.4 | 0 | 10±1.2 | 8±1 | 0 | 9±1.3 | 11±1.5 | 0 | 11±1.3 | 8±1.3 | 8±1.1 | X | 27±1.8 | 0 |

*S. aureus: Staphylococcus. aureus ATCC 25923; E. coli: Escherichia coli ATCC 25922; C. albicans: Candida albicans ATCC 1023, X±SD, mean of three mesearurements ± standard deviation*

C+_G_= Gentamicin, C+_N_= Nistatin, C-= DMSO 3%

**Supplementary Table 8**

The antibacterial and antifungal activity assay for the hybrid quinoline - imidazole/benzimidazole compounds **12a-k**, determined by [disk diffusion](https://www.sciencedirect.com/topics/biochemistry-genetics-and-molecular-biology/disk-diffusion) assay.

| **Strain** | **Compound / Diameter of inhibition zone (mm)** | | | | | | | | | | | | | |
| --- | --- | --- | --- | --- | --- | --- | --- | --- | --- | --- | --- | --- | --- | --- |
|  | **12a** | **12b** | **12c** | **12d** | **12e** | **12f** | **12g** | **12h** | **12i** | **12j** | **12k** | C+_G_ | C+_N_ | C- |
| *S. aureus* | 0 | 12±1.5 | 8±1.1 | 15±1.6 | 0 | 12±1 | 13±1.2 | 6±0.8 | 9±1 | 13±1.4 | 11±1 | 14±1.4 | X | 0 |
| *E. coli* | 0 | 12±1.2 | 20±2 | 20±1.8 | 0 | 24±1.7 | 10±1 | 13±1.3 | 13±1.2 | 14±1.2 | 10±1.1 | 12±1.1 | X | 0 |
| *C. albicans* | 0 | 0 | 0 | 0 | 0 | 0 | 0 | 0 | 0 | 0 | 0 | X | 27±1.8 | 0 |

*S. aureus: Staphylococcus. aureus ATCC 25923; E. coli: Escherichia coli ATCC 25922; C. albicans: Candida albicans ATCC 1023, X±SD, mean of three mesearurements ± standard deviation*

C+_G_= Gentamicin, C+_N_= Nistatin, C-= DMSO 3%

**Supplementary Table 9**

The minimum inhibitory concentration (MIC) for the hybrid quinoline - imidazole/benzimidazole compounds **7a-k**.

| **Strain** | **Compound / Minimum inhibitory concentration (MIC, μg/mL)** | | | | | | | | | | | | |
| --- | --- | --- | --- | --- | --- | --- | --- | --- | --- | --- | --- | --- | --- |
|  | **7a** | **7b** | **7c** | **7d** | **7e** | **7f** | **7g** | **7h** | **7i** | **7j** | **7k** | C+_G_ | C+_N_ |
| *S.aureus* | 0.312 | **0.039** | **0.09** | 0.156 | 0.625 | 0.156 | 0.625 | **0.078** | **0.039** | **0.078** | 0.19 | 0.5 | X |
| *E.coli* | 1.25 | 0.312 | 0.19 | 0.156 | 0.625 | 0.312 | 0.625 | **0.001** | **0.039** | **0.039** | 0.78 | 0.25 | X |
| *C.albicans* | 0 | 0.156 | 0.39 | 0.312 | 0 | 1.25 | 0 | 2.5 | 0.156 | 0 | 1.56 | X | 1.5 |

*S. aureus: Staphylococcus. aureus ATCC 25923; E. coli: Escherichia coli ATCC 25922; C. albicans: Candida albicans ATCC 1023;* C+_G_= Gentamicin, C+_N_= Nistatin

Bold values represent the good results

**Supplementary Table 10**

The minimum inhibitory concentration (MIC) for the hybrid quinoline - imidazole/benzimidazole compounds **8a-k**.

| **Strain** | **Compound / Minimum inhibitory concentration (MIC, μg/mL)** | | | | | | | | | | | | |
| --- | --- | --- | --- | --- | --- | --- | --- | --- | --- | --- | --- | --- | --- |
|  | **8a** | **8b** | **8c** | **8d** | **8e** | **8f** | **8g** | **8h** | **8i** | **8j** | **8k** | C+_G_ | C+_N_ |
| *S. aureus* | 0.312 | 0.625 | 0.156 | 2.5 | - | 5 | 0.312 | **0.019** | 0.156 | 1.25 | - | 0.5 | X |
| *E. coli* | 1.25 | 0.156 | 0.312 | 0.625 | - | 0.625 | **0.039** | **0.019** | **0.039** | 1.25 | 1.25 | 0.25 | X |
| *C. albicans* | - | - | - | - | - | - | - | - | - | - | - | X | 1.5 |

*S. aureus: Staphylococcus. aureus ATCC 25923; E. coli: Escherichia coli ATCC 25922; C. albicans: Candida albicans ATCC 1023,* C+_G_= Gentamicin, C+_N_= Nistatin

Bold values represent the good results

**Supplementary Table 11**

The minimum inhibitory concentration (MIC) for the hybrid quinoline - imidazole/benzimidazole compounds **11a-k**.

| **Strain** | **Compound / Minimum inhibitory concentration (MIC, μg/mL)** | | | | | | | | | | | | |
| --- | --- | --- | --- | --- | --- | --- | --- | --- | --- | --- | --- | --- | --- |
|  | **11a** | **11b** | **11c** | **11d** | **11e** | **11f** | **11g** | **11h** | **11i** | **11j** | **11k** | C+_G_ | C+_N_ |
| *S. aureus* | **0.078** | **0.078** | 0 | **0.039** | 0.156 | **0.039** | **0.009** | **0.00001** | **0.009** | **0.019** | 0.156 | 0.5 | X |
| *E. coli* | **0.039** | 0.156 | **0.039** | **0.039** | 0.625 | **0.009** | **0.019** | **0.004** | **0.019** | **0.039** | 0.156 | 0.25 | X |
| *C.albicans* | 0.312 | 0 | 0.156 | **0.078** | 0 | 0.312 | 0 | 0 | **0.039** | 0.312 | 0.625 | X | 1.5 |

*S. aureus: Staphylococcus. aureus ATCC 25923; E. coli: Escherichia coli ATCC 25922; C. albicans: Candida albicans ATCC 10231,* C+_G_= Gentamicin, C+_N_= Nistatin

Bold values represent the good results

**Supplementary Table 12**

The minimum inhibitory concentration (MIC) for the hybrid quinoline - imidazole/benzimidazole compounds **12a-k**.

| **Strain** | **Compound / Minimum inhibitory concentration (MIC, μg/mL)** | | | | | | | | | | | | |
| --- | --- | --- | --- | --- | --- | --- | --- | --- | --- | --- | --- | --- | --- |
|  | **12a** | **12b** | **12c** | **12d** | **12e** | **12f** | **12g** | **12h** | **12i** | **12j** | **12k** | C+_G_ | C+_N_ |
| *S. aureus* | - | 5 | 5 | **0.039** | - | 2.5 | 5 | **0.0003** | **0.019** | 0.625 | 0.156 | 0.5 | X |
| *E. coli* | - | **0.039** | **0.031** | **0.019** | - | 0.625 | **0.019** | **0.004** | **0.039** | 1.25 | **0.078** | 0.25 | X |
| *C. albicans* | - | - | - | - | - | - | - | - | - | - | - | X | 1.5 |

*S. aureus: Staphylococcus. aureus ATCC 25923; E. coli: Escherichia coli ATCC 25922; C. albicans: Candida albicans ATCC 1023,* C+_G_= Gentamicin, C+_N_= Nistatin

Bold values represent the good results

***NMR Spectrum of two QIBS representative compounds***

The NMR spectra were recorded on a Bruker Avance III 500 MHz spectrometer operating at 500 MHz for ^1^H and 125 MHz for ^13^C. The NMR apparatus is equipped with a 5 mm PABBO detection probe, and the program used for acquisition and processing data is TopSpin 3.2 PL5.

**Supplementary Figure 1**: ^1^H-NMR spectrum of representative compound **8c** (from **8a-k** series).

**Supplementary Figure 2**: ^13^C-NMR spectrum of representative compound **8c** (from **8a-k** series).

**Supplementary Figure 3**: ^1^H-NMR spectrum of representative compound **12h** (from **12a-k** series).

**Supplementary Figure 4**: ^13^C-NMR spectrum of representative compound **12h** (from **12a-k** series).
